# Circular RNAs in NSCLC: Their Role in Tumorigenesis, Tumor Microenvironment Reshaping, and Response to Therapies

**DOI:** 10.3390/cancers18182989

**Published:** 2026-09-16

**Authors:** Lea Cerato, Florence de Fraipont, Beatrice Eymin

**Affiliations:** 1 University Grenoble Alpes, INSERM U1209, CNRS UMR5309, Team RNA Splicing, Cell Signaling and Response to Therapies, Institute For Advanced Biosciences, F38000 Grenoble, France; 2 Medical Unit of Molecular Genetic (Hereditary Diseases and Oncology), Grenoble-Alpes University Hospital, F38000 Grenoble, France

**Keywords:** circular RNAs, NSCLC, therapy resistance, tumor immune microenvironment, biomarker

## Abstract

Lung cancer is the leading cause of cancer death worldwide. However, the molecular mechanisms involved in its development and response to therapies remain poorly understood. Previous studies have unraveled the pro- or anti-tumoral roles of circular RNAs (circRNAs) in numerous cancers. These mostly non-coding RNAs consist of a single-strand RNA forming a stable closed loop. CircRNA expression is often dysregulated in cancers, including lung cancer. An increasing number of studies have been published describing the role of circRNAs in lung tumor progression and/or resistance to therapies. Most of these studies have focused on how circRNAs impact cancer cells in a cell-autonomous manner, but less is known about the role of tumoral circRNAs in the crosstalk between cancer cells and cells from the tumor microenvironment (TME). In this review, focusing on non-small cell lung cancer (NSCLC), we provide a comprehensive and up-to-date analysis of the current knowledge in this emerging field. We highlight key molecular mechanisms by which tumor-derived circRNAs reshape the NSCLC tumor immune microenvironment. We also discuss recent findings showing their contributions to most NSCLC hallmarks, including those newly described in 2022, as well as their involvement in NSCLC’s escape from therapies. All these studies clearly open avenues for translational applications of circRNAs in NSCLC, as either potential biomarkers or therapeutic targets, although current limitations clearly need to be overcome to translate circRNAs into the clinic.

## Simple Summary

Circular RNAs (circRNAs) represent a novel and important class of endogenous, mainly non-coding RNAs, which result from pre-mRNA back-splicing. CircRNAs are now well-recognized as critical contributors to various cancer cell hallmarks in different types of cancer, including lung cancer. However, their involvement in the reshaping of the tumor microenvironment, more particularly the tumor immune microenvironment, and the development of resistance to therapies remains less explored, notably in non-small cell lung cancer (NSCLC). Here, we aim to provide a comprehensive and up-to-date review of the current knowledge in this still-emerging research field, as well as to discuss the potential of circRNAs as biomarkers for NSCLC detection and assessment, alongside the limitations associated with their clinical applications.

## 1. Introduction

Circular RNAs (circRNAs) are endogenous single-strand molecules that form covalently closed loops. They lack both a 5′ cap and 3′ polyadenylated tail but contain a characteristic 3′–5′ junction, known as the back-splicing junction (BSJ). Their circular structure confers them resistance to exonuclease degradation, making them more stable than linear RNAs [1]. CircRNAs were first identified by Sanger et al. in 1976, visualized by electron microscopy in a vegetal viroid [2]. For many years, circRNAs were regarded merely as byproducts of canonical splicing with no biological function [3]. However, advances in large-scale RNA sequencing and bioinformatics analyses have revealed their diversity among living organisms. CircRNAs have now been documented in numerous species across the eukaryotic evolutionary tree, including fungi (*Saccharomyces cerevisiae* and *Schizosaccharomyces pombe*); protists (*Plasmodium falciparum* and *Dictyostelium discoideum*) [4]; plants (*O. sativa*) [5]; and mammals such as rats, mice, and humans [6].

In the human genome, 6 to 23% of transcript genes give rise to circRNAs [7]. However, some circular RNAs are more expressed than their linear counterparts [8]. CircRNAs exhibit tissue-specific, cellular type-specific, and/or developmentally specific expression. For example, circRNAs are enriched in the brain and their expression is dependent on the stage of development as well as neuronal plasticity [9]. Within cells, circRNAs have been shown to regulate various cellular pathways by interacting with RNA-binding proteins (RBPs), microRNAs, or mRNAs. An increasing number of studies have shown that their expression is dysregulated in several diseases [10,11] including cancer [12]. The abnormal expression of many circular RNAs has been linked to tumor initiation, disease progression [13], and cancers’ resistance to certain treatments [14]. Additionally, circRNAs can be detected in various biological fluids such as urine, saliva, and blood; thus, they are extensively studied as potential biomarkers [15].

Lung cancer is the leading cause of cancer death worldwide, and circRNAs now appear to be critical contributors to lung tumor progression and response to treatment. Hence, numerous studies and excellent reviews have described the roles of dysregulated circRNAs on various hallmarks of lung cancer cells [16,17,18]. However, most of these studies have focused on how circRNAs impact cancer cells themselves in a cell-autonomous manner, while less is known about the role of tumoral circRNAs in the crosstalk between lung cancer cells and cells from the tumor microenvironment (TME). This is why, beside offering an up-to-date overview of the role of circular RNAs in lung cancer based on the cancer hallmarks newly described in 2022 [19], the main purpose of this review is to provide a comprehensive analysis of the current knowledge linking circRNAs and the reshaping of the lung tumor microenvironment, with a particular emphasis on the tumor immune microenvironment. We also discuss recent findings about the potential of circRNAs as biomarkers, alongside limitations associated with their clinical application. Notably, and because most of these studies have been performed in the non-small cell lung cancer (NSCLC) subtype, which represents 80–85% of all lung cancers, we have focused this review on this particular histological subtype. Hence, little is known about circRNAs’ involvement in small cell lung cancer (SCLC), although some studies have demonstrated their role in progression and chemotherapy resistance in this subtype [20,21].

## 2. Circular RNAs: Biogenesis and Main Functions

### 2.1. Circular RNA Biogenesis

Canonical splicing is a process that contributes to the maturation of pre-messenger RNAs into mature RNAs by removing introns and joining exons together, through the connection of a downstream 3′-splice site with an upstream 5′-splice site [22].

**Figure 1:**
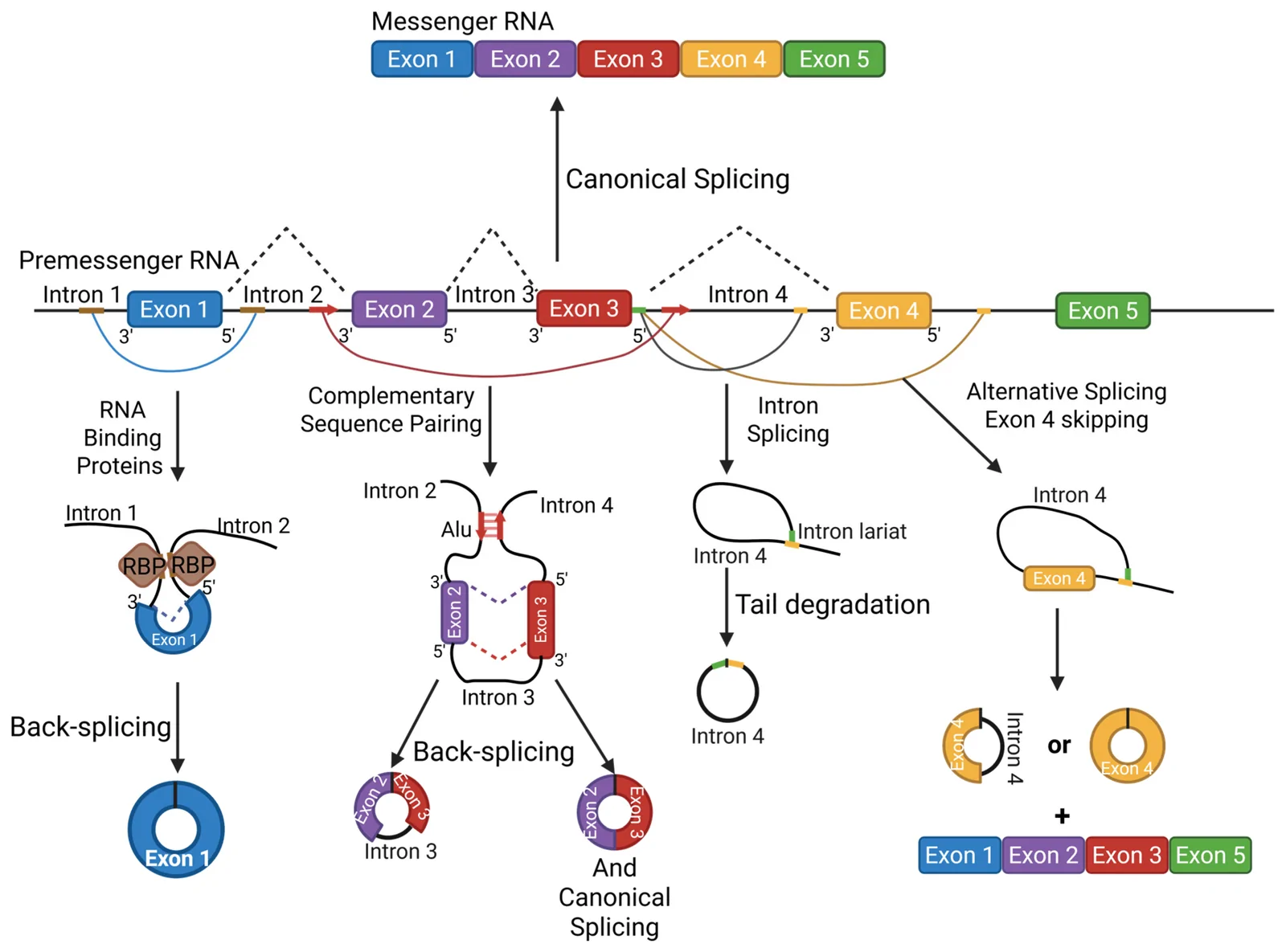
Biogenesis of circular RNAs. Schematic representation of the different back-splicing pathways involving RNA-binding proteins (RBPs), intronic complementary Alu sequences, and lariat circularization. Adapted from Qiu et al., Cancers, 2022 [29]. Created in Biorender. Cerato (2026) https://app.biorender.com/illustrations/68777953a8d96ebce2b64b43.

The particularity of circRNAs relies on their back-splicing junction, which connects an upstream 3′-splice site to a downstream 5′-splice site. Back-splicing can compete with canonical splicing. Three back-splicing pathways are demonstrably involved in circRNA biogenesis (Figure 1), and can generate three distinct types of circRNAs from the transcription of a single gene. The repeated reverse sequences involved in circRNA biogenesis are often the Alu sequence. Intron pairing at these Alu sequences facilitates physical proximity between specific donor 3′-splice sites and acceptor 5′-splice sites [23]. This process promotes exon circularization and leads to the synthesis of exonic circular RNAs (ecircRNAs) with exonic sequences only. This is responsible for the biogenesis of the majority of human circular RNAs [23]. Back-splicing can also be facilitated by trans-acting elements, such as RNA-binding proteins (RBPs) [24]. Certain RBPs possess double-stranded RNA-binding domains (dsRBDs) and interact with complementary intronic sequences, promoting exon circularization and stabilizing the back-splicing process [25]. RBPs lacking dsRBDs can also contribute to back-splicing by dimerizing upon binding to a specific intronic motif [25]. The back-splicing process is therefore tightly regulated by both cis-acting elements and trans-acting factors, which may function independently or in concert. It can produce ecircRNAs and exon–intron circular RNAs (elciRNAs) which retain both exonic and intronic sequences [26]. The final pathway involves exon skipping or lariat circularization. During canonical splicing, the intronic donor is cleaved from the exon, resulting in the formation of a lariat structure. During pre-messenger RNA maturation, specific exons may be excluded from the resultant transcript, a process known as alternative splicing [22]. Upon exclusion, an exon-containing lariat is generated, which, like the intronic lariat, is highly unstable and subject to degradation [27]. Circular intronic RNAs (ciRNAs) consist exclusively of intronic sequences, and elciRNAs can be generated from these lariats before their degradation via back-splicing, although the details of this mechanism remain unclear. In 2015, Kelly et al. showed a correlation between exon skipping and circularization, suggesting a link between alternative and back-splicing [28].

### 2.2. The Main Biological Functions of Circular RNAs

**Figure 2:**
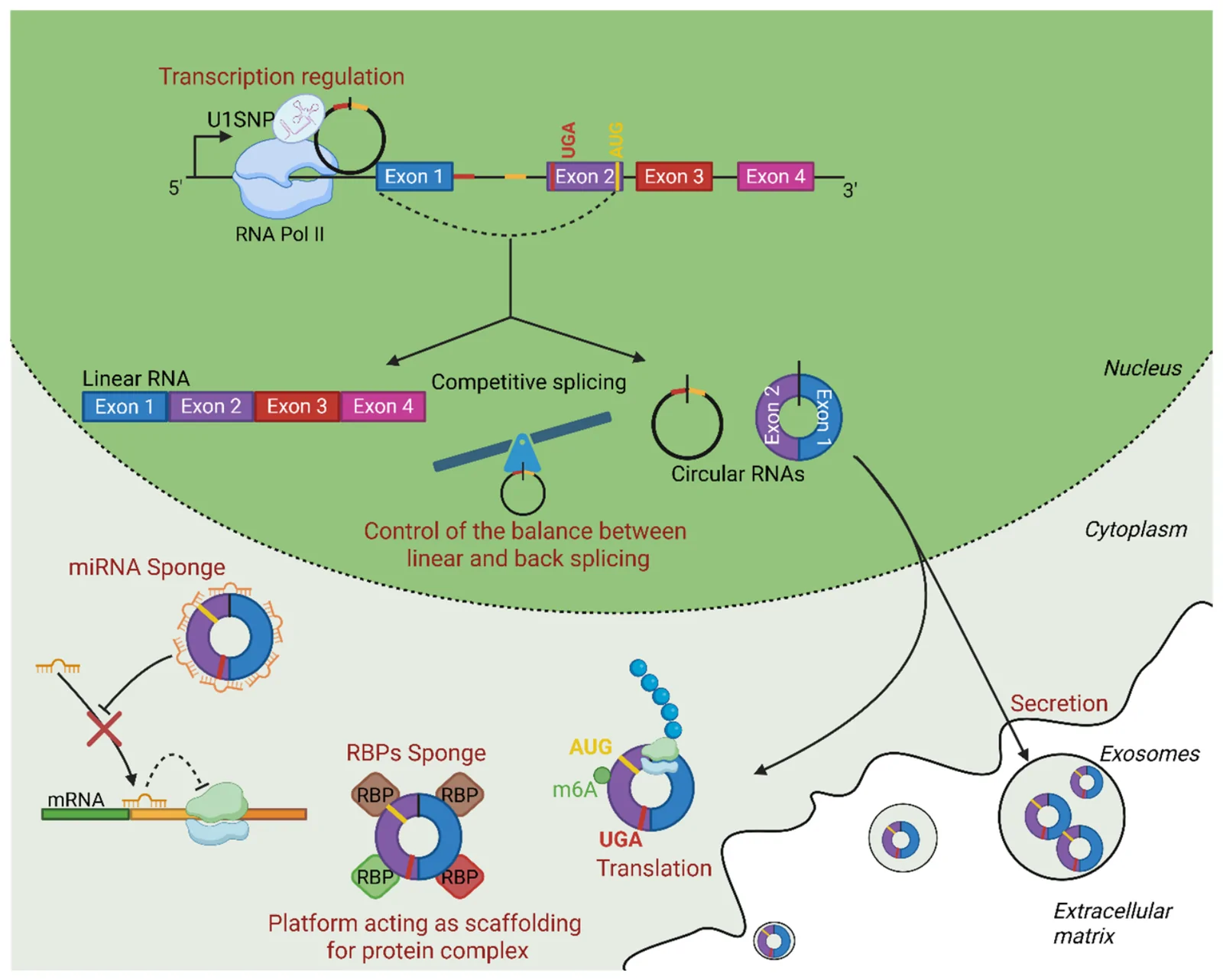
Biological roles of circular RNAs inside and outside the cell. Circular RNAs can interact with RNA polymerase II and the subunit U1SNP to regulate transcription in the nucleus, including that of their own genes; capture miRNAs and RNA-binding proteins (RBPs); be translated into proteins in the cytoplasm; and/or be secreted into the extracellular matrix, where they participate in inter-cellular communication. Created in Biorender. Cerato (2026) https://app.biorender.com/illustrations/68779fce9eb7120bb1fd305f.

The functions of circular RNAs in the cell depend on their sequence, post-transcriptional modifications, secondary structures, localization, and/or concentration. They have been described in detail in various reviews [16,17,18] and are summarized here (Figure 2).

#### 2.2.1. Regulation of Gene Expression

Circular RNAs present in the nucleus perform different functions in the regulation of gene expression. They can directly influence the expression of their own genes by regulating their transcription. CircRNAs can also inhibit their own transcription by forming three-stranded DNA:RNA hybrids known as R-loops at the parental locus, thereby slowing down transcription [30]. Additionally, nuclear elciRNAs can activate and modulate the transcription of other genes. They facilitate the recruitment of transcriptional regulatory proteins to the promoter region, forming a circRNA/DNA/protein complex [31].

Moreover, circRNAs can influence gene expression by forming circRNA/mRNA/protein complexes. For example, circZNF609 binds to various mRNAs, enhancing ELAVI protein recruitment, which stabilizes those mRNAs and boosts their translation [32]. Gene expression is also under the control of epigenetic modifications, and circRNAs can also regulate the transcription and recruitment of epigenetic regulators [33].

#### 2.2.2. Sponging of microRNAs (miRNAs)

Until now, the main function of cytoplasmic circRNAs was thought to be their ability to interact with miRNAs. miRNAs are small RNAs (17 to 25 nucleotides long) that bind to messenger RNAs through complementary sequences, thereby inhibiting their translation or tagging them for degradation. CircRNAs can possess sequences complementary with particular miRNAs. Therefore, by acting as sponges for miRNAs, circRNAs stabilize mRNAs and/or enhance their translation. As example, ciRS-7 contains more than 70 sites complementary to miR-7 [34]. Furthermore, ciRS-7 binds with great complementarity to miR-671, which induces the AGO2-dependent cleavage of ciRS-7, revealing the existence of feedback regulations between circRNAs and miRNAs [35].

Although the miRNA sponge function is commonly and largely attributed to numerous circRNAs, its validity is increasingly debated. One major issue is the stoichiometric imbalance. CircRNAs are typically scarce compared to abundant miRNAs in cells [36]. Some circRNAs overcome this by having multiple binding sites, though most circRNAs have only one or two per miRNA [37]. Other circRNAs target several miRNAs and influence the expression of various genes, expanding their cellular roles. Therefore, the bioinformatic prediction of circRNA–miRNA complementarity should be supplemented with spatial expression analysis within cells [38].

#### 2.2.3. Scaffolding Role: Interactions with Proteins, DNAs, and/or RNAs

CircRNAs have also been shown to interact with proteins, and to form complexes including circRNAs, proteins, and DNA or RNA.

Ashwal-Fluss et al. were the first to demonstrate an interaction between a circular RNA and a protein in 2014. They identified circMDL, which can bind to the protein MDL produced by the same gene. This protein also interacts with its own messenger RNA, encouraging its circularization. Therefore, a potential feedback loop exists: when the MDL protein is abundant, it suppresses the canonical splicing of its own mRNA and promotes the formation of circMDL; conversely, excessive circMDL binds the protein and inhibits its own back-splicing [39]. In addition, circular RNAs can serve as scaffolds for several proteins, promoting their interactions. As an example, circFOXO3 binds both the cyclin-CDK inhibitor p21^WAF1^ and CDK2, promoting p21^WAF1^ inhibition by CDK2 [40].

In addition, circular RNAs can assemble RNA/DNA promoter/protein complexes that facilitate gene transcription. The circRHOT1/*NRF2* promoter/TIP60 complex enhances *NR2F6* gene transcription by recruiting the acetyltransferase histone TIP60 [41].

#### 2.2.4. Encoding Functions

Historically, circular RNAs were considered non-coding RNAs. However, recent research has increasingly explored their translational potential. The translation of messenger RNAs is mostly cap-dependent. Unlike mRNAs, circRNAs lack both a polyadenylated tail and a 5′ cap, implying that their translation must be cap-independent. Currently, two distinct mechanisms of cap-independent translation have been identified: internal ribosome entry site (IRES)-driven translation and N6-methyladenosine-mediated translation.

IRESs are secondary or tertiary structures of RNA that can mediate the recruitment of the ribosomal 40S subunit to initiate translation [42]. It has been shown that introducing IRESs is sufficient to achieve efficient translation in eukaryotic cells [43]. Importantly, more and more circRNAs containing IRESs have been described and shown to be able to encode proteins [44,45]. In addition, the potential of circRNAs to encode neo-antigens in cancer has recently emerged [46], revealing circRNAs as an important source of neo-epitopes in cancer and, therefore, potentially interesting targets for anti-cancer immunotherapies and vaccine development [47].

Methylation at the sixth nitrogen atom of adenosine (m6A) constitutes a post-transcriptional modification frequently observed in circular RNAs [48]. The presence of m6A methylation on circular RNAs facilitates the recruitment of a translation initiation factor that engages the 40S ribosomal subunit, enabling the initiation of circular RNA translation [49]. Research conducted by Tang et al. in 2020 identified several hundred peptides encoded by circular RNAs in male germ cells, with their translation appearing to be dependent on m6A modifications within the sequence [50].

During translation, the ribosome synthesizes amino acids from the RNA sequence, initiating at the start codon and ending at the stop codon. Certain circular RNAs possess a start codon yet lack a stop codon, resulting in infinite open reading frames. In such instances, rolling circle translation can yield proteins of exceptionally high molecular weight [51].

#### 2.2.5. Triggering Inter-Cellular Communication

Circular RNAs can be secreted from the cell into the extracellular matrix either as free circulating RNAs or inside exosomes or other extracellular vesicles. Indeed, it has been shown that circular RNAs can be enriched in exosomes compared to the producing cell [14]. These findings are promising for biomarker development using circular RNA quantification in liquid biopsies (see III. II. Detection of circRNAs in liquid biopsies), but they also suggest a role for circular RNAs in inter-cellular communication. For example, tumor cells can secrete circular RNAs that influence all other cell types surrounding them; for example, circUSP7 is secreted by non-small cell lung cancer cells and regulates cytotoxic T cells [52]. Conversely, cells from the tumor microenvironment can communicate with cancer cells by secreting circRNAs, such as circEIF3K, which is derived from fibroblasts and transferred to colorectal cancer cells [53].

## 3. CircRNAs in NSCLC

Lung cancer is the leading cause of cancer death worldwide, causing more than 1.7 million deaths in 2018. At the national level, 52.777 new cases were diagnosed in 2023 and lung cancer was responsible for 30.400 deaths in 2021, with a 5-year survival rate of 20% (National Cancer Institute, 2024 [54]). Lung cancer is an extremely heterogeneous pathology that can develop from different sites of the bronchopulmonary system. Two main entities can be identified: small cell lung cancer (SCLC) and non-small cell lung cancer (NSCLC). The latter accounts for approximately 85% of lung cancers and comprises two major subtypes: squamous cell carcinoma and non-squamous cell carcinoma, including adenocarcinoma, the most prevalent histological subtype. One-third of patients with NSCLC have a locally advanced tumor at diagnosis that cannot be surgically resected. Although advances in tumor genomics have revolutionized patient care, particularly with the identification of specific molecular abnormalities that allow patients to be treated with targeted therapies that are more effective than conventional chemotherapies [*Epidermal Growth Factor Receptor* (EGFR)-activating mutations, *Anaplastic Lymphoma Kinase* (ALK) gene rearrangement], only a limited number of patients can benefit from these new therapies. For the majority of patients, cytotoxic chemotherapy based on platinum salts in combination with immunotherapies is preferred. However, a large proportion of patients have primary resistance or acquire secondary resistance to these treatments. In recent years, an increasing number of studies have unraveled the role of circRNAs in lung tumorigenesis, which involves both cell-autonomous and non-cell-autonomous mechanisms, as well as in lung tumor escape from various therapies. Because most of these studies have been performed in NSCLC, we decided to focus this review on this subtype.

### 3.1. Role of circRNAs in Lung Tumorigenesis: Cell Autonomous Effects

**Figure 3:**
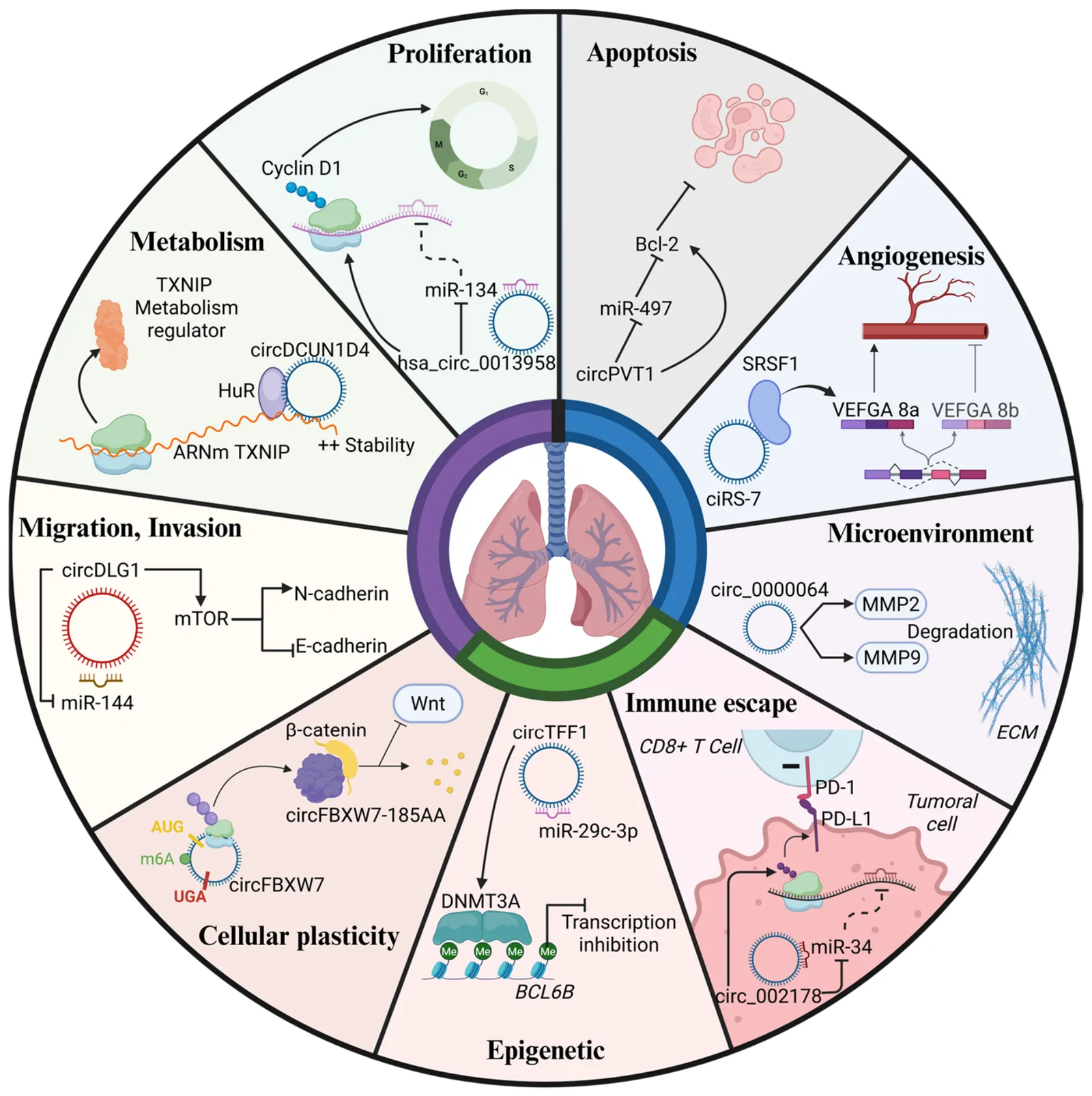
Role of circular RNAs during the tumorigenic process: 9 of the 14 described hallmarks are represented with an example of a circular RNA and the mechanism by which it acts in lung cancer. Created in Biorender. Cerato (2026) https://app.biorender.com/illustrations/687bb287cdd3b789f2084260.

As cancer cells transform, they develop traits and phenotypes that set them apart from normal cells. These unique features, referred to as “cancer hallmarks” by Hanahan et al., now total 14, with four new hallmarks identified in 2022 [19]. In NSCLC, the dysregulation of circular RNA expression has been linked to nine of these hallmarks (Figure 3).

#### 3.1.1. Role in Proliferation of Lung Cancer Cells

Cell division is a highly regulated process controlled by several factors, such as cyclin-dependent kinases (CDKs). Cell cycle disruptions in cancer cells lead to uncontrolled and aberrant proliferation, a crucial phenomenon in cancer initiation and progression. Hsa_circ_0013958 is found at high levels in both the tissues and plasma of lung adenocarcinoma patients. It functions as a sponge by capturing miR-134, which leads to increased translation of the *Cyclin D1* oncogene. *Cyclin D1* is essential for cell cycle progression, and when overexpressed, it enhances tumor cell proliferation and invasive potential [55]. In addition, the circRACGAP1/miR-144-5p/CDKL1 axis promotes CDKL1 translation in pulmonary cancer cells where circRACGAP1 is abundant [56]. CDKL1, a cyclin-dependent kinase required for the G2-to-mitosis transition, increases cell proliferation when its presence is elevated in tumor cells. CircITCH is underexpressed in lung cancers [57]. According to Wan et al., circITCH binds to two miRNAs, miR-7 and miR-214, and controls the translation of the protein ITCH encoded by the same gene. ITCH is a ubiquitinase that regulates the Wnt/β-catenin signaling pathway by promoting the degradation of phosphorylated DVL2, an important regulator of this pathway [58]. Wnt/β-catenin inhibition decreases the cell proliferation capacity upon the expression of circITCH. Circular RNAs present in exosomes also participate in cell cycle regulation. CircERBB2IP secreted in exosomes is transferred to neighboring cells and regulates the expression of the oncogene *PSAT1*, by capturing miR-519-3p, leading to tumor growth in mouse models [59]. Recently, circLIMK1-005 has been reported to be overexpressed in LUAD patients. CircLIMK1-005 activates the CDK4 pathway by increasing the expression of the Cyclin D1 and CDK4 proteins. The mechanism involved is a binding of circLIMK1 to the protein RPA1 and the latter’s stabilization, which promotes Cyclin D1 and CDK4 accumulation [60].

#### 3.1.2. Role in Apoptosis

Resistance to apoptosis, or programmed cell death, is very important during tumor development. Apoptosis is regulated by pro- and anti-apoptotic factors that are often dysregulated in cancers. High expression of circPVT1 is associated with a poor prognosis in NSCLC. CircPVT1 inhibits miR-497 and indirectly increases the anti-apoptotic factor Bcl-2 [61]. Bcl-2 is also regulated by cyclin D1, itself inhibited by miR-326, which binds to circPUM1. CircPUM1 inhibition in lung adenocarcinoma cells decreases proliferation, migration, and invasion while increasing apoptosis [62]. Hsa_circ_0020123 possesses binding sites for multiple miRNAs, including miR-144 in lung cancer. The capture of this miRNA results in increased expression of ZEB1 and EZH2, two critical proteins implicated in cancer growth and metastasis development [63], owing to their role in gene regulation. ZEB1 is a transcription factor, and EZH2 is a methyltransferase. Additionally, hsa_circ_0020123 can bind and capture miR-193a-3p, which regulates IRF4, an immune response regulator capable of activating the NOTCH-AKT pathway [64] and thereby promoting glycolysis and proliferation, and inhibiting apoptosis in NSCLC cells [65]. Furthermore, the interaction of hsa_circ_0020123 with miR-512-3p increases the expression of CORO1C, a protein dysregulated in several cancers that contributes to increased proliferation, migration, invasion, and angiogenesis and inhibits apoptosis [66].

#### 3.1.3. Role in Invasion and Metastasis Processes

Epithelial-to-mesenchymal transition (EMT) allows cancer cells to acquire greater migratory capacities, to resist some treatments, and to create metastases distant from the primary tumor site by colonizing other tissue types. EMT requires the reprogramming of cellular gene expression, which relies on the inhibition of genes associated with epithelial features (e.g., *CDH1,* which codes for E-cadherin) and an increase in mesenchymal genes (e.g., *N-cadherin*). This transition can be initiated by various stimuli, such as TGF-β. TGF-β-induced EMT is inhibited by circPTK2 when it is overexpressed in lung cancer, via the uptake of miR-429 and miR-200b-3p [67]. Numerous signaling pathways, including PI3K, MAPK, and WNT/β-catenin, are involved in the migration and invasion of tumor cells [68]. CircDLG1 regulates the AKT/mTOR pathway via miR-144 capture. CircDLG1 overexpression leads to an increase in mTOR expression in NSCLC cells, which increases mesenchymal markers such as N-cadherin while decreasing epithelial markers such as E-cadherin [69]. Zhao et al. showed a role for circVAPA in the proliferation, migration, invasion, and stem cell phenotype of NSCLC cells through the regulation of the WNT/β-catenin pathway. CircVAPA sponges miR-876-5p and releases its target WNT5A, a member of the WNT protein family, leading to WNT/β-catenin pathway activation [70]. The MAPK/ERK pathway regulates circC190 expression in NSCLC. The overexpression of circC190 leads to ERK1/2 phosphorylation, which stimulates cell proliferation and migration in murine xenografts [71].

#### 3.1.4. Role in Metabolism Rewiring

Imperfect vascularization of the tumor leads to oxygen and nutrient deficiencies. To compensate, cancer cells adapt and alter their energy metabolism, making glycolysis their primary energy source; this is known as the Warburg effect [72]. Glycolysis consists of a series of enzymatic reactions that break down glucose into two pyruvates. This metabolic pathway is relatively inefficient but can occur aerobically or anaerobically. The ENO1 protein is a glycolytic enzyme involved in this pathway. Its gene encodes a circular RNA, circ-ENO1, which regulates its own gene by inhibiting miR-22-3p. The combined overexpression of the ENO1 protein and circ-ENO1 in lung adenocarcinoma tissues promotes glycolysis and tumor progression [73]. CircZNF707 overexpression in NSCLC tissues also enhances cancer cells’ glycolysis through the circZNF707/miR-668-3p/PFKM axis. PFKM is a vital enzyme in the glycolytic energy metabolism pathway [74]. CircDCUN1D4 is a circular RNA that serves as a biomarker of survival in lung cancer. It acts as a scaffold by binding directly to the HuR protein and the TXNIP mRNA, thereby promoting their interaction. This circDCUN1D4/HuR/TXNIP mRNA complex increases the stability of the mRNA and the translation of the protein it encodes. The TXNIP protein regulates cellular metabolism, and its expression in cancer decreases glycolysis and metastasis [75].

#### 3.1.5. Role in Cancer Cell Plasticity

Cancer cell plasticity is represented by the cell’s ability to dedifferentiate, avoid differentiation, or transform into another cell lineage. This plasticity allows cancer cells to modulate their rate of proliferation or migration. It was proposed as an emerging cancer hallmark in 2022, highlighting its importance in cancer development. Stem cells are highly plastic: they proliferate and self-renew, but they can also generate specialized daughter cells. Cancer stem cells (CSCs) possess the same characteristics. They are a subpopulation of cancer cells that differ from other tumor cells by their dedifferentiation; slow proliferation; expression of certain pluripotent transcription factors, such as SOX2, NANOG, and OCT4; expression of enzymes such as ALDH1A1; and expression of surface markers like CD44, CD24, and CD133. This population is known to exhibit resistance to chemotherapy and radiotherapy. It also gives rise to metastases and initiates tumors in xenografts [76]. The inhibition of hsa_circ_0003222 in NSCLC cells reduces the stem cell phenotype. This circular RNA binds to miR-527, leading to increased expression of PHF21B, a stem cell marker. The overexpression of hsa_circ_0003222 increases the expression of proteins associated with the stem cell phenotype, such as CD44, CD133, OCT4, and SOX2 [77]. Li et al. studied resistance to EGFR-TKIs in patients with EGFR-mutant lung adenocarcinoma, as well as the maintenance of cancer stem cell renewal via the WNT signaling pathway. They demonstrated that the WNT pathway is regulated by a peptide translated from circFBXW7. This protein, named circFBXW7-185AA, interacts with β-catenin, reducing its stability and, consequently, the activation of the WNT pathway. The overexpression of circFBXW7 inhibits, via its regulation of the WNT pathway, the self-renewal of cancer stem cells and promotes asymmetric division, resulting in differentiated cells. CircFBXW7 therefore reduces the stem cell phenotype and resistance to EGFR-TKI-targeted therapies [78].

#### 3.1.6. Role in Epigenetic Reprogramming

Epigenetics describes changes in genes’ activity that are not linked to mutations in their sequences. DNA methylation is an epigenetic mechanism that has been identified as promoting the development of several cancers [79]. The methyltransferase DNMT3A is regulated by miR-29c-3p, whose inhibition is linked to circTFF1. The overexpression of circTFF1 in lung cancer cell lines leads to the overexpression of DNMT3A and strong methylation of the *BCL6B* gene, while its inhibition increases cell proliferation, migration, and invasion [80]. Shen et al. demonstrated an overexpression of circNOTCH1, which binds competitively to METTL14, thereby preventing the binding of the methyltransferase enzyme complex to the NOTCH1 messenger RNA. The latter, being unmethylated, is more stable and its translation is more efficient. The NOTCH1 protein increases the proliferative capacity of lung cancer cells [81]. Hsa_circ_0077837, overexpressed in NSCLC patients and predicting poor survival, increases the methylation of *PTEN*, which downregulates its expression. The low expression of *PTEN* results in the inhibition of apoptosis in NSCLC cells [82].

### 3.2. Role of circRNAs in Lung Tumorigenesis: Non-Cell-Autonomous Effects

In addition to impacting various features of lung cancer cells themselves, circRNAs also contribute to non-cell-autonomous effects which greatly reshape the tumor microenvironment (TME). The TME consists of various cell types (e.g., endothelial cells, fibroblasts, immune cells) and the extracellular matrix (ECM). Each component plays a role in tumor initiation, progression, and resistance to treatments.

#### 3.2.1. Remodeling of Extracellular Matrix (ECM)

The extracellular matrix (ECM), produced by all cells within the microenvironment, provides essential mechanical support as well as a reservoir of signaling molecules. It is composed of components such as fibronectin, collagen, proteoglycans, and polysaccharides; both the quantity and degree of cross-linking among these elements contribute to tissue rigidity. The ECM serves as a physical barrier, protecting tumor cells from therapeutic interventions and restricting cellular migration. Circular RNAs participate in the remodeling of the ECM. For instance, circ_0000064 regulates the matrix-degrading metalloproteinases MMP2 and MMP9 in lung cancer [83].

#### 3.2.2. Regulation of Angiogenesis

Angiogenesis is the process by which new blood vessels form from an existing one, and it plays a key role during tumor progression. Angiogenesis is initiated and regulated by several factors, including vascular endothelial growth factor A (VEGF-A). The VEGF-A receptor is located on endothelial cells. Through alternative splicing and the selection of a differential alternative splicing site in exon 8, *VEGF-A* pre-messenger RNA can give rise to two isoforms, VEGF-A_165_a and VEGF-A_165_b, which are pro- and anti-angiogenic, respectively [84]. SRSF1 is a splicing factor whose overexpression in lung cancer cells can be stimulated by particles smaller than 2.5 µm and that can promote the expression of the VEGF-A_165_a isoform. CiRS-7 binds to the serine- and arginine-rich sequence of SRSF1 and inhibits its ubiquitination and degradation. Thus, through an RNA-binding protein-sponging mechanism, ciRS-7 increases the cellular abundance of SRSF1 and promotes the expression of the pro-angiogenic isoform VEGFA_165_a [85]. The blood supply to tumor cells is often defective, leading to hypoxic stress within the tumor. To compensate for this lack of oxygen, cancer cells express the transcription factor HIF-1. The HIF-1α subunit is involved in proliferation, survival, and angiogenesis. CircPIP5K1A is a tumor promoter. It regulates the expression of HIF-α by inhibiting miR-600 [86], thereby increasing proliferation and metastasis in NSCLC.

#### 3.2.3. Remodeling of the Tumor Immune Microenvironment (TIME)

The tumor immune microenvironment (TIME) determines a tumor’s immunophenotype, and includes immune cells that contribute to innate and/or adaptive immunity. Innate immunity detects pathogens in the body in order to destroy them. It also activates adaptive immunity, involving T and B lymphocytes, by presenting them with pathogen antigens. The infiltration of immune cells into tumor regions; their location, density, and type; and their pro-inflammatory and anti-tumoral functions or, conversely, immunosuppressive and pro-tumoral functions allow us to define the so-called tumor immune “landscape”. Based on this “landscape”, a classification system called the Immunoscore has been developed [87]. This is a prognostic assessment tool that relies on quantifying the immune cells infiltrating the tumor core or the peripheral invasive zone. The TIME is finely regulated by numerous factors originating from all the cells that compose it. Importantly, circular RNAs are also messengers that participate in the regulation of this TIME.

##### Regulation of Tumor Infiltration by Immune Cells

**Figure 4:**
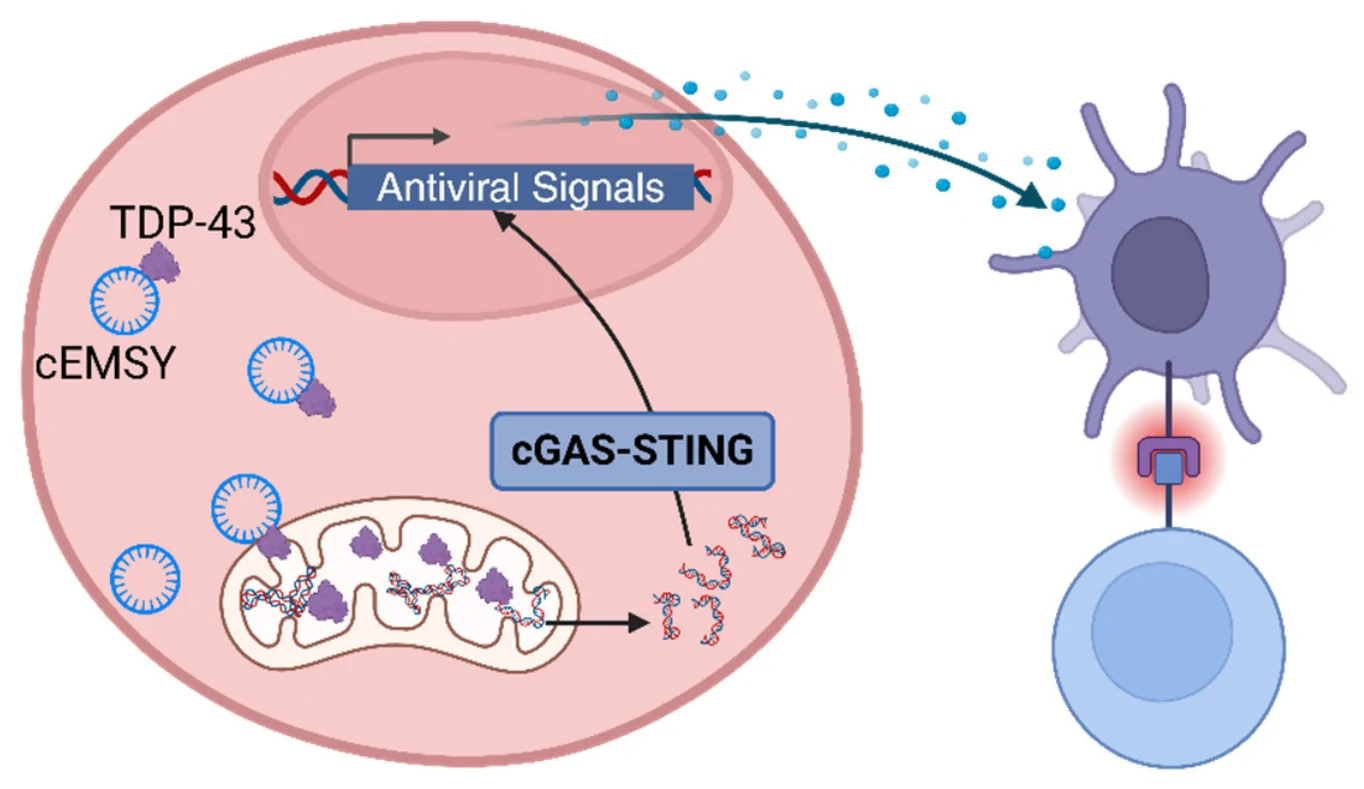
Role of cEMSY circular RNA in cancer cells and its influence on dendritic cells. Created in Biorender. Cerato (2026) https://app.biorender.com/illustrations/689f4d6d92de59444fdbb81b.

Some studies have shown that circular RNAs remodel the TIME by regulating the infiltration of immune cells into the tumor. For example, the expression of circCDR1as has been associated with the degree of immune and stromal cell infiltration. Its high expression correlates with a low density of CD8+ T cells (*p* < 0.001) and active NK cells (*p* < 0.001), and enhanced infiltration of monocytes (*p* < 0.001) and neutrophils (*p* = 0.002). High CDR1as expression is found in patients with a high pro-tumoral M2 macrophage polarization ratio (*p* < 0.001) [88]. The relationship between CDR1as expression and the immune infiltrate relies on its role in the TGF-β signaling pathway and in the control of gene expression related to the interaction of extracellular matrix receptors, as well as the composition and organization of the extracellular matrix. The HOXC9 protein, overexpressed in lung cancer, is correlated with a poor prognosis. This protein is regulated by circCENPF, which inhibits hsa-miR-184. Furthermore, its expression is correlated with the infiltration of CD8+ T cells (*p* < 0.0001), CD4+ T cells (*p* < 0.05), macrophages (*p* < 0.05), and dendritic cells (*p* < 0.05), as well as with the expression of certain immune cell markers, such as ROS1 and COX2 for M1 macrophages, CD163 for M2 macrophages, and CD141 and DC1C for dendritic cells. HOXC9 is also associated with the expression of T cell exhaustion markers, such as PD-1 [89]. Circ_BBS9, which is underexpressed in lung adenocarcinoma, is a tumor suppressor. It plays a role in cell proliferation and ferroptosis. Circ_BBS9 inhibits miR-7150 and leads to increased expression of the IFIT3 protein. The IFIT3 protein is positively correlated with the infiltration of immune cells, including neutrophils, CD8+ and CD4+ T lymphocytes, macrophages, and dendritic cells (*p* < 0.0001 for all cell types). IFIT3 expression is also correlated with the expression of several immunostimulators such as CD80, CD86, and ICOS (*p* < 0.0001) as well as with the production of chemokines such as CXCL11, CXCL10, and CCL8 (*p* < 0.0001) [90]. Moreover, IFIT3 is associated with the infiltration of regulatory Treg cells (*p* < 0.0001) and with the expression of various immune modulators, such as PDCD1LG2, CD274, and HAVCR2 (*p* < 0.0001). Thus, via the IFIT3 protein, circ_BBS9 modulates the composition of the tumor immune microenvironment [90]. The recruitment of CD8+ T lymphocytes and dendritic cells is also affected by the expression of circNDUFB2 by cancer cells. The overexpression of this circular RNA increases the secretion of CXCL10, CXCL11, CCL5, and IFN-β. Because these cytokines are key players in the immune response, their regulation by circNDUFB2 influences the infiltration of DCs and T cells into the tumor microenvironment [91]. The transcription of CXCL10 and CXCL11 is also regulated by the circRNA circFNDC3B, which is upregulated in NSCLC tissues. CircFNDC3B binds to the protein TFII-I and disturbs its interaction with STAT1, resulting in the inhibition of CXCL10 and CXCL11 transcription. The consequence of circFNDC3B overexpression is the impairment of CD8+ T cell infiltration in the tumor [92]. In lung cancer, the circular RNA cEMSY expressed by cancer cells allows the aggregation of the TDP-43 protein in mitochondria, leading to the leakage of mitochondrial DNA into the cytoplasm. This leakage activates the cGAS-STING pathway, which induces the expression of antiviral signals. These signals increase the cross-presentation of CD8+ T lymphocytes by dendritic cells. High cEMSY expression is associated with greater infiltration of dendritic cells and CD8+ T lymphocytes in lung adenocarcinoma. cEMSY is also a biomarker of a favorable response to immunotherapies (Figure 4) [93].

##### Regulation of the Expression of Co-Inhibitory Molecules

**Figure 5:**
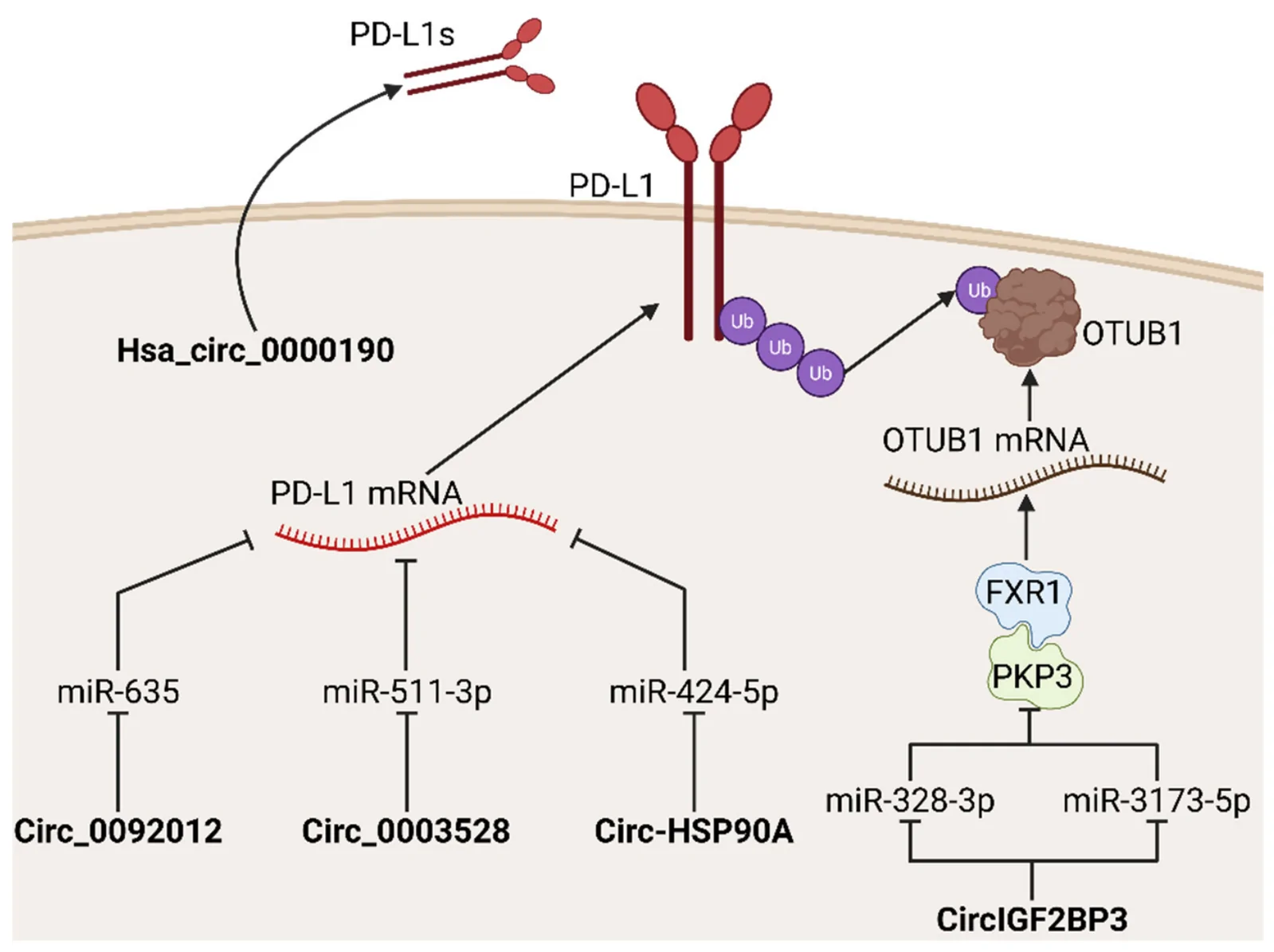
Various circular RNAs are involved in the expression of membranous or soluble PD-L1 by cancer cells via the capture of miRNAs, leading to the expression of the PD-L1 messenger RNA or the expression of the OTUB1 deubiquitinase protein, which prevents the degradation of PD-L1. Hsa_circ_0000190 is linked to the expression of soluble PD-L1 (PD-L1s). Created in Biorender. Cerato (2026) https://app.biorender.com/illustrations/68c275f001b7d4eed9260b6d.

The immune system (IS) can detect and eliminate cancer cells to prevent tumor initiation and growth; this is called immunosurveillance. To evade this surveillance, cancer cells have developed defense mechanisms, such as expressing immune-inhibitory receptors on their surfaces and secreting cytokines, chemokines, and other soluble factors that promote an immunosuppressive environment. Numerous circular RNAs have been described as modifying the interaction between cancer cells and T lymphocytes. Mechanistically, these circular RNAs regulate the expression of co-inhibitory molecules and thereby affect T cell activity and viability as well as cytokine secretion (Figure 5) [94]. CircCHST15 is an example of a circular RNA overexpressed in lung cancer that participates in tumor cell evasion from immune system recognition. It inhibits miR-155-5p and miR-194-5p, leading to the overexpression of PD-L1, which binds to PD-1 present on the membranes of immune cells. Their interaction inhibits the proliferation and function of immune cells [95]. Furthermore, circCHST15 regulates the expression of certain cytokines and chemokines, such as CCL17, CCL22, IFN-γ, TNF-β, and IL-10. These soluble factors present in the tumor microenvironment play a key role in the immune response and activation. CircRNA-002178 also regulates the expression of PD-L1 in lung cancer cells. This circular RNA is overexpressed in tissues, serum exosomes, and cancer cells, where it binds to miR-34, an inhibitor of PD-L1. The overexpression of circRNA-002178 and PD-L1 induces the depletion of cytotoxic T lymphocytes responsible for the destruction of cancer cells [96]. The PD-1/PD-L1 axis promotes CD8+ T cell apoptosis and thereby immune escape in NSCLC cells overexpressing circ_001678. This circular RNA activates the PD-1/PD-L1 pathway by promoting the production of the protein ZEB1. The mechanism involved is a capture of miR-326, a ZEB1 inhibitor [97]. Hsa_circ_0000190 regulates the expression of PD-L1 and CD80 on the surface of NSCLC-derived cancer cells, and its overexpression increases the secretion of soluble PD-L1. Consequently, hsa_circ_0000190 decreases T cell activation. Patients respond better to immunotherapy when their soluble PD-L1 concentration is low [98]. Inhibiting this circular RNA “in vivo” in a murine model of lung cancer resistant to anti-PD-L1 immunotherapy decreased tumor growth. The circular RNA circ-HSP90A originates from the gene encoding heat shock protein 90A (HSP90A) and is overexpressed in NSCLC. It captures miR-424-5p and enables PD-L1 expression. Circ-HSP90A stimulates CD8+ T cell apoptosis through the interaction of the immune inhibitors PD-L1/PD-1 (*p* < 0.01) [99]. CircIGF2BP3 captures miR-328-3p and miR-3173-5p, enabling PKP3 expression. A negative correlation between PKP3 expression and the pro-inflammatory chemokine CCL5 has been observed. Furthermore, PKP3 binds to FXR1 to stabilize OTUB1, a deubiquitinase that inhibits PD-L1 degradation. Through this mechanism, circIGF2BP3 reduces CD8+ T cell infiltration into the tissues of NSCLC patients and suppresses the T cell response “in vitro” and “in vivo” [100]. The prognostic factor circ_0003528 is overexpressed in NSCLC tissues and cell lines. It plays a role in resistance to apoptosis, viability, proliferation, epithelial–mesenchymal transition, invasion, and angiogenesis. Its role in immune evasion relies on the regulation of PD-L1 expression through the inhibition of miR-511-3p [101]. Circ_0092012 is a diagnostic biomarker overexpressed in NSCLC that correlates with tumor size, TNM stage, and lymph node metastases [102]. Its inhibition in cancer cells decreases their viability, proliferation, resistance to apoptosis, and migratory and invasive capacities. Furthermore, it reduces immune escape. The apoptosis of CD8+ T cells after co-culture with cancer cells decreases upon the inhibition of Circ_0092012. Their production of IFN-γ and TNF-α increases under the same conditions, indicating increased CD8+ T cell activity. PD-L1 expression is regulated in cancer cells by miR-635, which is itself inhibited by Circ_0092012. This regulatory pathway increases the immune escape of cancer cells and tumor growth “in vivo” [102].

##### Regulation of Macrophages’ Polarization

Tumor-associated macrophages (TAMs) adopt M1 and M2 phenotypes. Zhang et al. investigated whether the expression profile of circular RNAs could distinguish these two phenotypes. Using microarrays of polarized bone marrow-derived macrophages, they characterized 189 differentially expressed circular RNAs; among these, circRNA-003780, circRNA-010056, and circRNA-010231 were overexpressed, while circRNA-003424, circRNA-013630, circRNA-001489, and circRNA-018127 were inhibited (*p* < 0.05) in the M1 phenotype compared to the M2 phenotype [103]. CircHIPK3 and circPTK2 are overexpressed in KRAS-mutated lung cancer tissues. They are also overexpressed in metastatic tissues relative to the primary tumor. They are present in serum exosomes from patients and overexpressed in exosomes from those with metastases. Both are also overexpressed in tissues rich in M2 macrophages, such as the stroma. They trigger the infiltration of M2-polarized macrophages, leading to immunosuppression and promoting tumor growth. CircHIPK3 is primarily expressed in the M1 subtype, while circPTK2 is expressed in the M2 subtype. Inhibiting circPTK2 in macrophages reduces tumor size and the number of metastases, and improves survival in mice [104].

**Figure 6:**
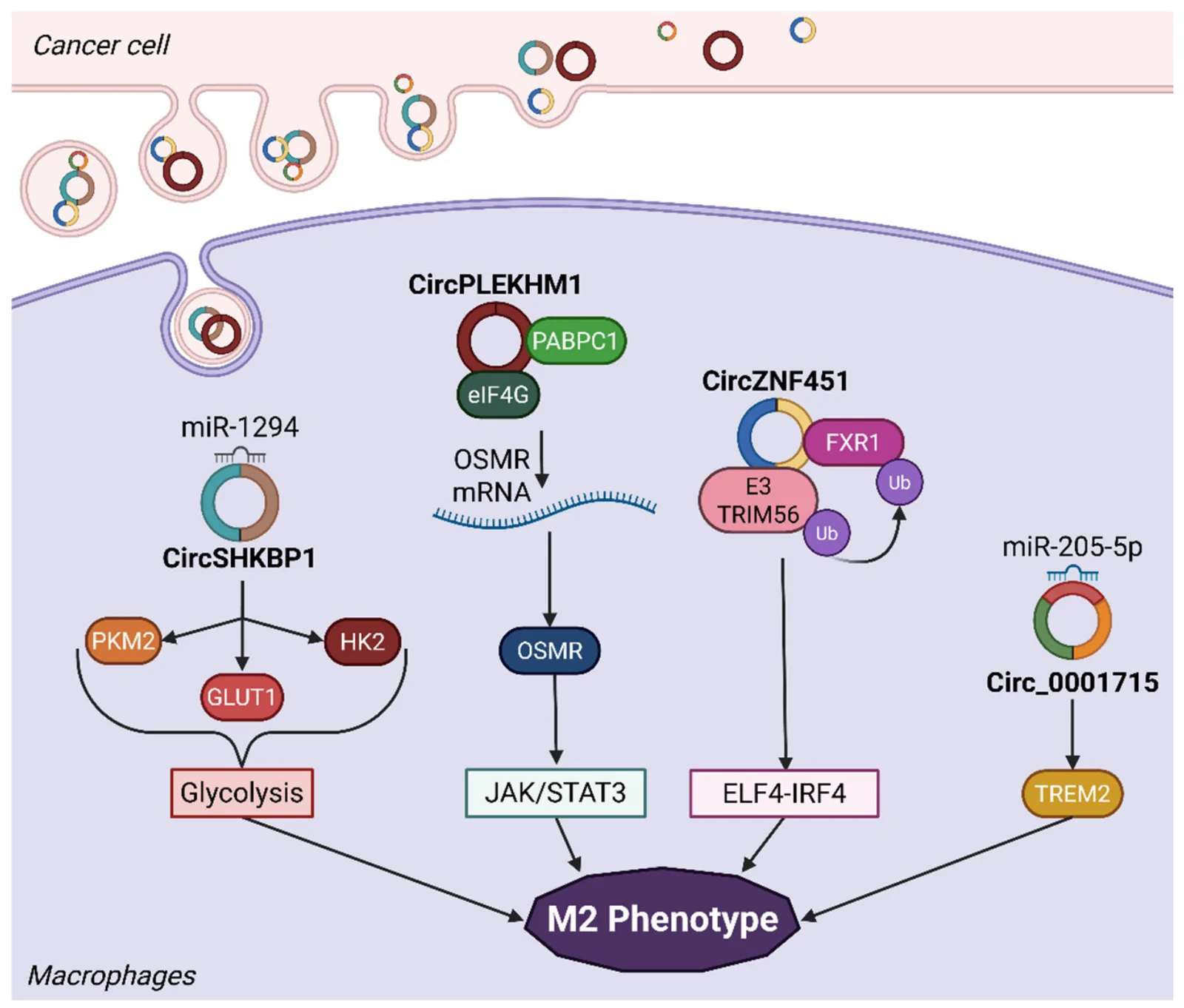
Signaling pathways regulated by circular RNAs derived from cancer cells that promote an M2 pro-tumoral phenotype in macrophages. Created in Biorender. Cerato (2026) https://app.biorender.com/illustrations/68c29657b5ac4d884a867f35.

Tumor-derived circular RNAs can also influence the phenotype of tumor-associated macrophages via their transfer from one cell type to another. Figure 6 shows some examples of circular RNAs produced by cancer cells and transferred to macrophages, where they induce M2-like polarization. In turn, pro-tumoral M2 TAMs contribute to cancer progression. CircSHKBP1 is overexpressed in tissues and serum exosomes from patients with NSCLC. In cancer cells, circSHKBP1 captures miR-1294 and regulates the expression of PKM2, an enzyme that catalyzes the progression of glycolysis. Through this pathway, circSHKBP1 increases the proliferation, migration, invasion, stem cell phenotype, and glycolysis of cancer cells. “In vitro”, culturing macrophage precursors in the presence of exosomes derived from cancer cells expressing circSHKBP1 increases M2 polarization markers such as CD206, IL-10, and IL-4, and decreases the expression of M1 polarization markers such as IL-12 and TNF-α. CircSHKBP1 transferred to macrophages via exosomes inhibits miR-1294 and promotes the expression of the PKM2, HK2, and GLUT1 proteins, all involved in glycolysis, thereby inducing the metabolic reprogramming of macrophages and M2 polarization. “In vivo”, exosomal circSHKBP1 promotes macrophage infiltration into the tumor microenvironment (*p* < 0.01) [105]. CircZNF451, secreted into exosomes, modulates the immune tumor microenvironment. Patients with high expression of circZNF451 show decreased infiltration of CD8+ T cells and M1 macrophages, while the infiltration of Treg cells and M2-polarized macrophages is increased. Upon transfer to macrophages, circZNF451 binds to the E3 ubiquitin ligase TRIM56 and FXR1, thereby promoting FXR1 ubiquitination. FXR1 degradation activates the ELF4-IRF4 pathway in macrophages, enhancing their anti-inflammatory M2 phenotype [106]. CircZNF451 expression is associated with a poor prognosis in patients with lung adenocarcinoma. Furthermore, circZNF451 is overexpressed in patients who progress compared to those with partial remission after anti-PD-1 immunotherapy. Hypoxia is a major characteristic of most tumors. This condition affects the expression of numerous genes. The hypoxia-inducing factor HIF1a induces the expression of circPLEKHM1 in NSCLC cells, which is then secreted into exosomes. Macrophages in the microenvironment incorporate circPLEKHM1 via exosome internalization. In these cells, it binds to eIF4G and PABPC1, enabling their interaction. This complex stabilizes OSMR’s mRNA and promotes its translation. This protein activates the JAK/STAT3 signaling pathway, which induces the M2 polarization of macrophages [107]. The TREM2 protein, overexpressed in lung adenocarcinoma cells, promotes proliferation and metastasis. In macrophages, this protein is regulated by miR-205-5p, which is itself inhibited by circ_0001715, and promotes M2 polarization. This polarization increases the viability, number of clones, migration, and invasion of NSCLC cells [108]. Finally, circATP9A packed in exosomes and transported to macrophages promotes their M2 polarization. In NSCLC cells, circATP9A’s packaging is favored by hnRNPA2B1, which recognizes the specific motifs GGAG/CCCU present in the circRNA sequence. Once absorbed by the macrophage, circATP9A induces an M2 pro-tumoral phenotype through a mechanism which remains to be elucidated [109].

##### Regulation of Dendritic Cells

Dendritic cells (DCs) are immune cells that detect and integrate pathogens. They are antigen-presenting cells and can therefore activate the adaptative immune system. In cancer, they can detect the tumorous cells, activate T lymphocytes, and participate in their recruitment to the cancer site. Despite the critical roles played by DCs in the TIME, to date, very few studies have been published concerning the molecular mechanisms by which circular RNAs expressed by cancer cells impact the maturation and/or immunophenotypes of the different subtypes of dendritic cells. Some studies have described the role of circRNAs in regulating dendritic cell maturation. The GDF15 protein induces a tolerogenic state in dendritic cells when overexpressed. Its overexpression leads to the inhibition of the circular RNA MALAT-1 and the NF-κB pathway in these same cells [110]. This study revealed a correlation between the expression of the circular RNA MALAT-1 and dendritic cell function. Furthermore, this circular RNA is overexpressed in mature dendritic cells compared to immature ones [110]. CircSnx5 suppresses DC activation by binding to miR-544, thereby enabling the expression of the SOCS1 protein, which in turn regulates DCs’ function and activation [111]. CircAEBP2 and circFSCN1 are both overexpressed in mature DCs compared to immature DCs. Their inhibition in DCs decreases T cell activation, increases Treg generation, alters cytokine production by dendritic cells, and decreases the expression of mature DC markers such as MHC class II and the co-stimulatory molecules C40 and CD80. The role of circFSCN1 in DCs’ maturation appears to involve the phosphorylation of RelAp65, a major component of the NF-κB pathway, which is decreased with circular RNA inhibition, while FOXO3 phosphorylation increases under the same conditions. CircFSCN1 expression is regulated by the immunosuppressive cytokines TGF-β and GDF15 [112]. CircAEBP2 also modulates the NF-κB pathway; its inhibition decreases the phosphorylation of the p65 protein. CircAEBP2 binds to the hnRNP protein and regulates the hnRNP/phosphorylated p65 signaling pathway, enabling the development of mature DCs [113].

### 3.3. CircRNAs as Key Contributors of Lung Tumor Resistance to Therapies

CircRNAs have been shown to be involved in the resistance of lung cancer cells to various therapies, including platinum salts, targeted therapies [e.g., EGFR-tyrosine kinase inhibitors (EGFR-TKIs) or anaplastic lymphoma kinase (ALK) inhibitors], or immunotherapies targeting the PD-L1/PD-1 axis.

#### 3.3.1. Resistance to Platinum Salts

**Figure 7:**
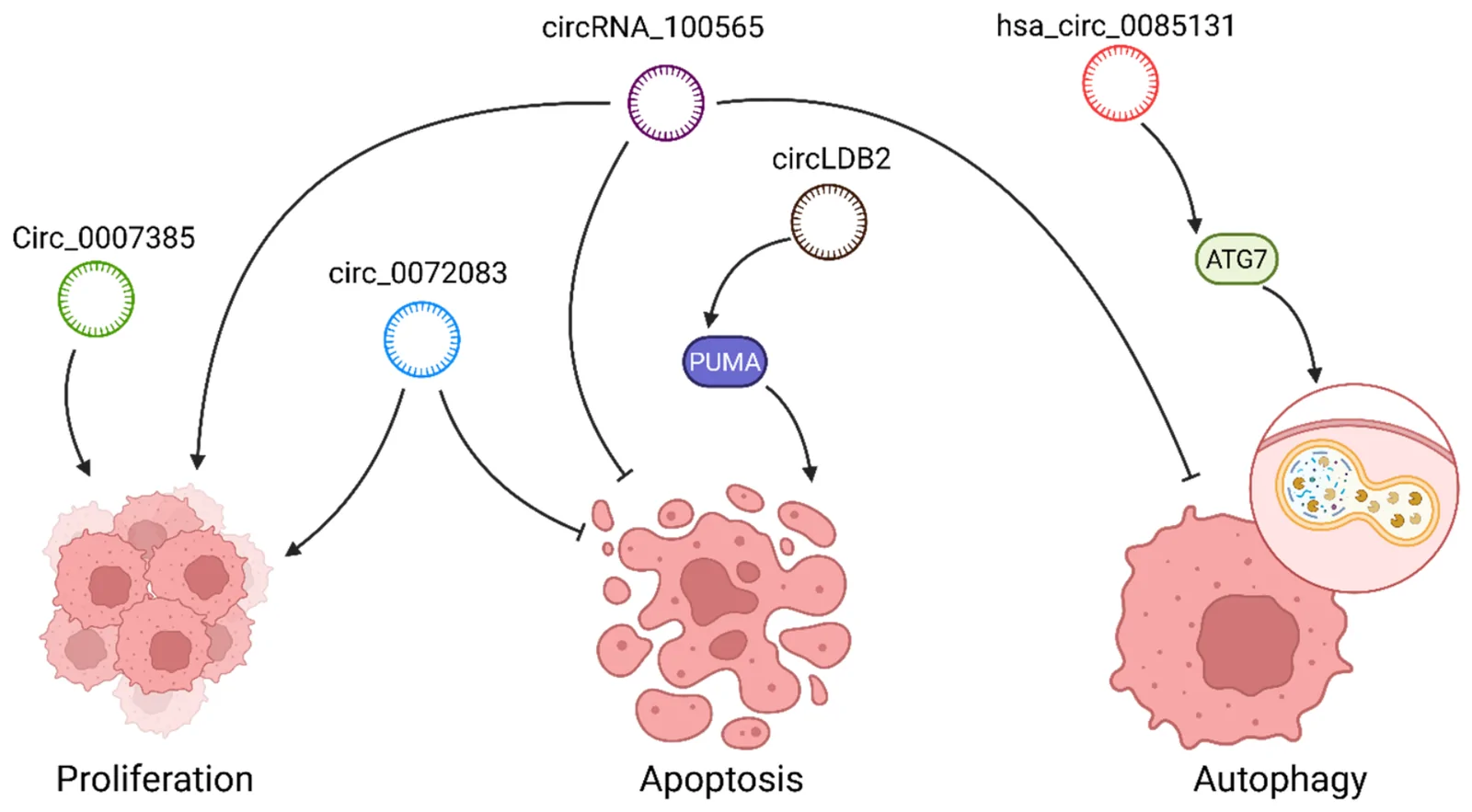
Examples of circular RNAs involved in cisplatin resistance due to their effects on the proliferation, apoptosis, or autophagy of lung cancer cells. Created in Biorender. Cerato (2026) https://app.biorender.com/illustrations/68b9af6c417c25455687e6d8.

**Figure 8:**
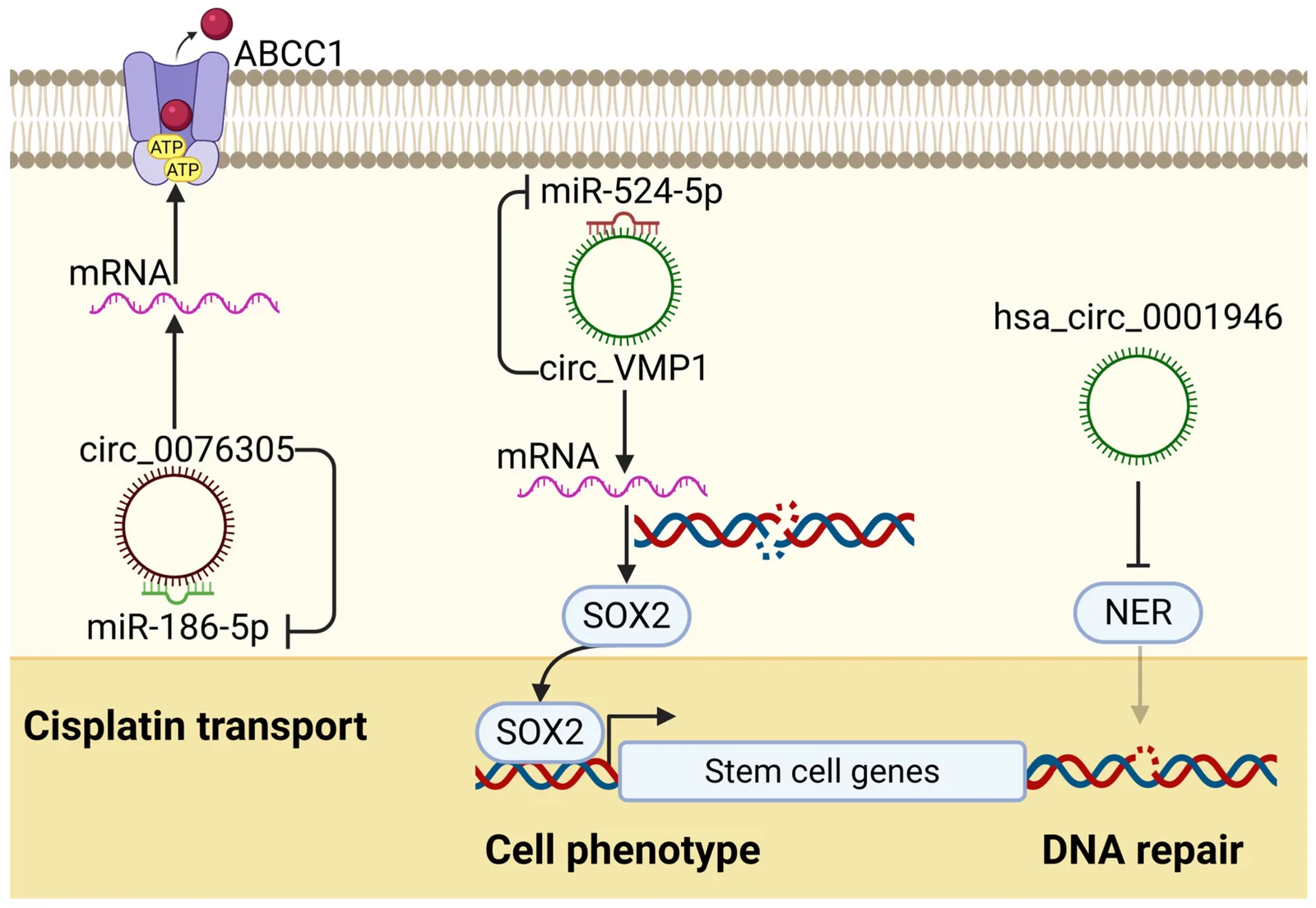
Roles of circular RNAs in resistance to platinum-based (cisplatin) chemotherapy in lung cancer through three distinct mechanisms impacting the transport of the molecule, the modulation of the tumor cell phenotype, and the repair of DNA damage. Created in Biorender. Cerato (2026) https://app.biorender.com/illustrations/68b9a14664d26e15084d3172.

Platinum-salts, alone or in combination with immunotherapies, have become one of the gold standard therapies in advanced NSCLC patients without targetable mutations. Numerous circRNAs have been identified as being involved in platinum salt resistance in lung cancer through different mechanisms (Figure 7 and Figure 8, Table 1) [114].

##### Effects on Cellular Proliferation

Circ_0072083, overexpressed in lung cancer, binds to miR-545-3p and regulates CBLL1. This protein, an E3 ubiquitinase ligase, facilitates the growth and motility of NSCLC cells [157]. Inhibiting circ_0072083 promotes apoptosis and inhibits the proliferation of cisplatin-treated cells [117]. Circ_0007385, whose overexpression is correlated with decreased overall survival in patients, also plays a role in cisplatin resistance by promoting cell proliferation and motility. The molecular mechanism involved is the inhibition of miR-519d-3p, which leads to the overexpression of HMGB1, a protein that promotes proliferation [118]. The overexpression of circ_PIP5K1A observed in cisplatin-resistant NSCLC tissues increases the proliferation, cell cycle progression, and mobility of NSCLC cells. It acts as sponge for miR-493-5p and allows the translation of the oncoprotein ROCK1 [119]. Circ_0010235 induces cisplatin resistance in NSCLC by increasing the production of E2F7, a protein with huge importance in cell cycle control, by inhibiting miR-379-5p [120].

##### Effects on Cellular Autophagy

The expression of hsa_circ_0085131 is higher in lung tumor tissues than in healthy tissues. Its overexpression is associated with a higher relapse rate and lower overall survival. Hsa_circ_0085131 contributes to cisplatin resistance by regulating cell autophagy [121]. Autophagy is a self-digestion process essential for cell survival, enabling the degradation of proteins and organelles. It is also an important cell death process in several pathologies, such as cancer [158]. Through its recycling action within the cell, autophagy protects cells from destruction caused by cisplatin treatment. Platinum-resistant NSCLC tumor cells overexpress hsa_circ_0085131, which binds to miR-654-5p and allows the expression of the autophagy-involved factor ATG7. This results in low expression of the autophagy inhibitor p62 and high expression of the autophagy-positive marker LC3-II [121].

##### Effects on Cellular Apoptosis

Cisplatin damages cellular DNA, leading to cell death by apoptosis. The overexpression of circRNA_100565 has been demonstrated in tissues and cell lines exhibiting cisplatin resistance. Its neutralization reduces cisplatin resistance in NSCLC cells by attenuating their proliferation and promoting apoptosis and autophagy. CircRNA_100565 regulates the miR-337-3p/ADAM28 pathway, through which it appears to inhibit cell death [122]. Conversely, circLDB2 increases sensitivity to cisplatin in non-squamous lung carcinoma cells. It allows the expression of the LIMCH1 protein by capturing its inhibitor miR-346. LIMCH1 is an anti-tumoral protein that upregulates the expression of the pro-apoptotic protein PUMA. The circLDB2/miR-346/LIMCH1 pathway promotes the anti-tumor effect of cisplatin and cell apoptosis [123].

##### Effects on Cellular Migration

The SNAIL protein is a transcription factor that largely contributes to the EMT process, thereby enhancing migration. This transcription factor is regulated by the FXR1/PRCKI complex, which promotes its accumulation. Circ_0000079, which is underexpressed in cisplatin-resistant NSCLC patients, inhibits the formation of this complex by interacting with FXR1, thereby hindering EMT. Circ_0000079 therefore inhibits cell invasion and counters cisplatin resistance [124]. CircHUWE1, downregulated in resistant tissues and cells, reduces their migratory and invasive capacity, viability, and ability to form colonies. It induces cisplatin resistance via the miR-34a-5p/TNFAIP8 pathway [125]. The overexpression of circ_0004015 in NSCLC cells promotes cisplatin resistance. It enhances KLF8 production via inhibiting miR-198 and increases the migration and invasion capacity of the cells, as well as their proliferation and apoptosis resistance [126].

##### Effects on Cellular Metabolism

Cells can modulate their own metabolism to reduce their sensitivity to cisplatin by using glycolysis as an energy source. Circ_0110498 regulates glycolysis in NSCLC cells and promotes their resistance to cisplatin. It captures miR-1287-5p and allows the expression of RBBP4. RBBP4 has been identified as a diagnostic marker and a marker of poor prognosis in lung cancer [159]. Inhibiting circ_0110498 inhibits tumor growth, prevents metastasis, decreases cellular glycolysis, and increases cell sensitivity to cisplatin [128]. Furthermore, hsa_circ_103809 is overexpressed in cisplatin-resistant NSCLC cells compared to sensitive cells, and the resistance of the latter increases with the level of circular RNA expression. Hsa_circ_103809’s role in resistance consists of inhibiting miR-377-3p and increasing the expression of the GOT1 protein, a crucial regulator of glutamate metabolism [160].

##### Effects on ABC Transporters

These transporter proteins are involved in the influx of cisplatin molecules into the cell. Their expression therefore plays a crucial role in resistance. In the case of resistance to cisplatin chemotherapy, Wang et al. demonstrated the overexpression of circ_0076305 in exosomes, tissues, and cell lines. The role of circ_0076305 in resistance is based on its ability to regulate the expression of ABCC1, which is itself regulated by miR-186-5p [129]. Another transporter, the ABCB1 protein, is also implicated in cisplatin resistance in lung cancer patients. ABCB1’s expression is regulated by circ_103615, which is itself overexpressed in patient tissues and cisplatin-resistant cell lines [130]. The molecular mechanism responsible for this regulation is not yet understood.

##### Regulation of Cancer Stem Cell Phenotype

The phenotype of cancer stem cells involved in resistance to various chemotherapeutic treatments is regulated by circCDR1as in NSCLC cells. CircCDR1as increases the expression of the HOXA9 protein by inhibiting miR-641. In cells overexpressing circCDR1as and HOXA9, the expression of the cancer stem cell phenotype markers SOX2, OCT4, and NANOG is increased, which correlates with cisplatin resistance [161]. SOX2, an oncogenic protein linked to various cancers [162], is regulated by circVMP1 via the inhibition of miR-524-5p. CircVMP1 is overexpressed in serum exosomes from chemotherapy-resistant patients compared to sensitive patients. Exosomes containing circVMP1 can thus transmit resistance to sensitive tumor cells through the regulation of the miR-524-5p/SOX2 axis [131].

##### Regulation of DNA Repair Pathways

Hsa_circ_0001946 can modulate the nucleotide excision repair (NER) signaling pathway in lung cancer cells, thereby increasing sensitivity to chemotherapy [132]. Its inhibition in cancer cells is correlated with an increase in the expression of proteins involved in NER, such as XPA, XPC, and ERCC1.

#### 3.3.2. Resistance to Targeted Therapies

##### Resistance to EGFR-TKIs

**Figure 9:**
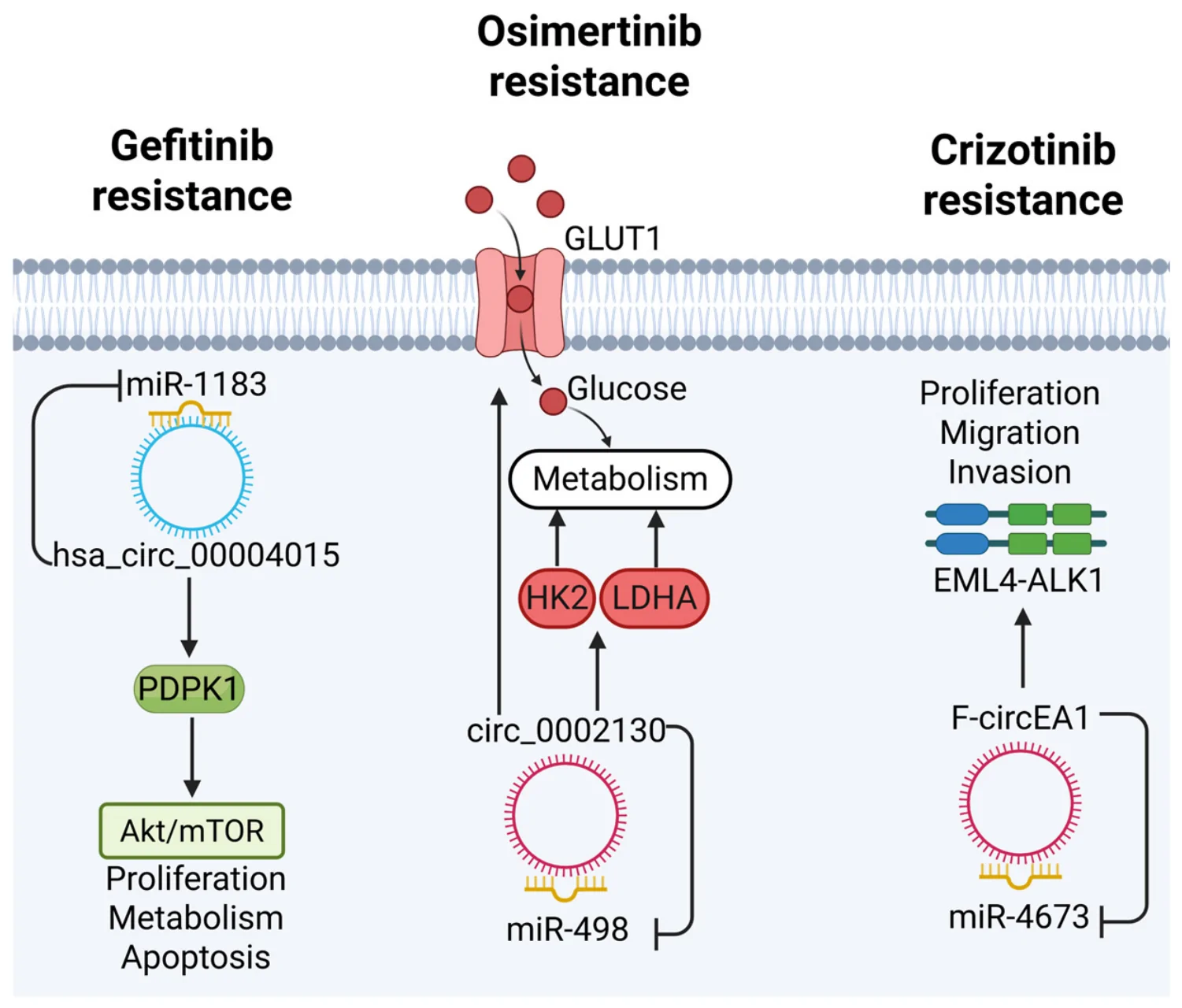
Roles of three selected circular RNAs in resistance to targeted therapies in lung cancer. Hsa_circ_00004015 and circ_0002130 are involved in resistance to therapies targeting the *EGFR* mutation and F-circEA1 in resistance to therapies inhibiting EML4-ALK1. Created in Biorender. Cerato (2026) https://app.biorender.com/illustrations/687d1db35a697ade428a3aac.

CircRNA_102481 is highly expressed in the exosomes of patients resistant to gefitinib, a first-generation EGFR-TKI. CircRNA_102481 regulates miR-30a-5p, thereby increasing the expression of the tumor promoter *ROR1*. Inhibiting this circular RNA restores sensitivity to gefitinib [133]. Another example is hsa_circ_0004015, which is overexpressed in cancerous tissues and associated with a poor prognosis. Hsa_circ_0004015 regulates the miR-1183/PDPK1 axis, which affects resistance to gefitinib (Figure 9). The PDPK1 protein is a component of the AKT-mTOR pathway, which plays a role in proliferation, metabolism, and apoptosis [127]. By regulating the ABCG2 protein, circSETD3 contributes to acquired resistance to gefitinib. By inhibiting miR-520h, circSETD3 overexpression increases ABCG2 production and the efflux transport of gefitinib [134]. Ma et al. demonstrated that resistance to osimertinib, the lead compound of third-generation EGFR-TKIs widely used in the clinic, is associated with increased glycolysis. They also showed that proteins involved in glycolysis—GLUT1, a glucose transporter, and HK2 and LDHA, which participate in glucose metabolism—are targets of miR-498, which is inhibited by circ_0002130. The overexpression of this circular RNA promotes resistance to osimertinib. Circ_0002130 is also overexpressed in exosomes purified from the serum of patients exhibiting resistance to osimertinib [135]. On the other hand, inhibiting hsa_circ_0005576 in osimertinib-resistant lung adenocarcinoma cells decreases proliferation and increases apoptosis. The mechanism of action of hsa_circ_0005576 is based on the capture of miR-512-5p, which targets IGF1R. This receptor is important for maintaining homeostasis in the cell [163]. Through analyzing microarray datasets, hsa_circ_0078465 has recently been identified to play a role in osimertinib resistance via the regulation of miR-183-5p [136]. CircSPINT2 has been identified to be downregulated in osimertinib-resistant cell lines. Its overexpression sensitizes cells to osimertinib by increasing RBP1 protein expression through the sponging of miR-1296-3p [137].

##### Resistance to ALK Inhibitors

A few circular RNAs have been reported as being involved in resistance to therapies targeting the EML4-ALK (echinoderm microtubule-associated protein-like 4-anaplastic lymphoma kinase) fusion protein. The circular RNA F-circEA1 is a fusion RNA derived from the *EML4-ALK* fusion gene. The overexpression of this circular RNA in various lung cancer cell lines, both expressing and lacking the *EML4-ALK* fusion, increases cancer cell proliferation, migration, and invasion. Its inhibition increases cell sensitivity to crizotinib, an ALK inhibitor, by decreasing the expression of the EML4-ALK1 protein and downstream signaling pathways, such as mTOR, JAK3-STAT3, and MEK-ERK1/2 [138]. F-circEA1 acts by capturing miR-4673, which can bind to the promoter of the *EML4-ALK1* gene [139].

#### 3.3.3. Resistance to Anti-PD-L1/PD-1 Immunotherapies

**Figure 10:**
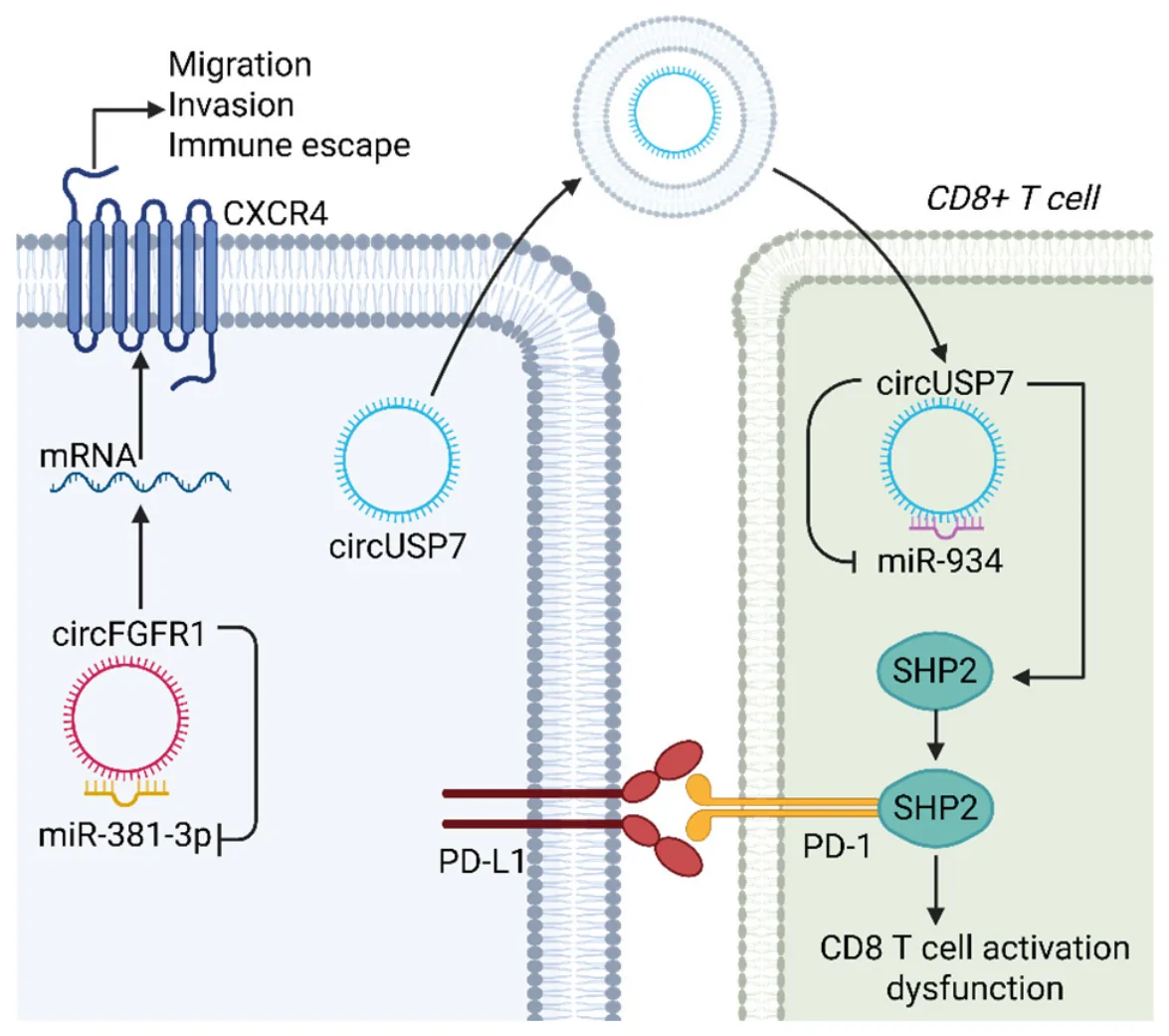
Two examples of circular RNAs involved in the resistance to immunotherapy in lung cancer. Created in Biorender. Cerato (2026) https://app.biorender.com/illustrations/6a63424c7a1abfd955a8b2c0.

Circular RNAs influence the response to anti-PD-L1/PD-1 antibodies through several mechanisms (Figure 10). CircFGFR1 is overexpressed in lung cancer tissues and promotes migration, invasion, and escape from anti-PD-L1 therapies by promoting the expression of the CXCR4 chemokine receptor [140]. Through the CXCR4/SDF-1 interaction, this receptor allows the recruitment of immune system cells, specifically plasmacytoid dendritic cells. The recruited cells exhibit an altered function that suppresses the cytotoxic activity of T lymphocytes, preventing them from eliminating cancer cells [164]. In lung cancer, circFGR1 regulates CXCR4 by inhibiting miR-381-3p [140]. The overexpression of circUSP7 in lung cancer cell exosomes is associated with a poor prognosis and CD8+ T cell dysfunction, leading to resistance to anti-PD-1 therapies. These T cells are normally activated by antigen presentation on the cancer cell. CircUSP7 is secreted by tumor cells and internalized by T cells. In these cells, it inhibits miR-934, thereby increasing the expression of SRC homologous region 2 (SHP2), a protein tyrosine phosphatase 2 recruited during PD-L1/PD-1 binding between cancer cells and T cells. SHP2 inactivates signaling induced by T cell receptors (TCRs) that detect the antigen. The overexpression of this protein in T cells caused by circUSP7 therefore leads to dysfunction of the anti-tumoral immune response. The overexpression of circUSP7 promotes NSCLC’s progression and resistance to anti-PD-1 [52]. Circ_CELF1 is overexpressed in NSCLC patients and is associated with malignant characteristics and a poor outcome. It is also linked to anti-PD-1 resistance. By inhibiting miR-491-5p, it increases the expression of the oncogene *EGFR*, leading to the progression of NSCLC and its resistance to immunotherapy [141]. In NSCLC tissues, the overexpression of circHMGB2 is an indicator of poor prognosis for patients and is associated with the exhaustion of CD8+ T, natural killer, and dendritic cells. This circular RNA captures miR-181a-5p and increases the production of CARM1, which is an inhibitor of the type 1 interferon response and whose overexpression is linked decreased efficacy of anti-PD-1 therapy [142]. Circulating extracellular vesicles containing circ_PPAPDC1A promote resistance to immune checkpoint inhibitors (ICIs) in NSCLC cells. Circ_PPAPDC1A promotes NSCLC progression “in vivo” and is a biomarker of diagnostic and shorter progression-free survival. This circular RNA promotes the proliferation of NSCLC cells and inhibits their apoptosis. It acts in ICI resistance by inducing the exhaustion of CD8+ T cells [143].

## 4. CircRNAs as Biomarkers in Lung Cancer

A biomarker is a precisely measurable biological parameter that reflects a patho-physiological state. Biomarkers are used in cancer research to help in diagnosis and prognosis, as well as in treatment selection or response. Interestingly, numerous studies have now revealed the potential of circular RNAs as biomarkers in NSCLC (Table 1). This increasing number of studies has been made possible by the development of new technologies enabling the detection and quantification of circRNAs in various clinical samples [165]. Hence, massive parallel sequencing techniques have revolutionized the study of circular RNAs. Algorithms have been specifically developed to improve the detection and quantification of circular RNAs [166]. One approach, based on the generation of libraries of back-splicing junctions, is reliable for detecting and quantifying circular RNAs of interest, but does not allow for the detection of new circular RNAs [165]. A second method allows for the alignment of back-splicing junctions with the genomic sequence, enabling the discovery of new circular RNAs, but is less precise.

The first techniques used to detect and quantify circular RNAs were reverse transcription (RT)–polymerase chain reaction (PCR) and quantitative RT-qPCR using primers positioned on each side of the back-splicing junction and in a divergent manner; they cannot amplify linear RNA from the same gene. Digital droplet PCR (ddPCR) is based on the same principle as PCR but is more sensitive than PCR and qPCR, allowing the absolute quantification of RNA molecules [165]. These methods require knowledge about the circular RNA sequence in order to define suitable primers and allow for the analysis of only one circRNA at a time. Recently, multiparametric analyses such as Nanostring nCounter have been adapted to the analysis of circRNAs and the identification of circRNA signatures [154,156].

### 4.1. Detection of circRNAs in Tissue Biopsies

Several studies have analyzed the transcriptomes of lung cancer tissues in comparison to healthy tissues to identify dysregulated circular RNAs that could be useful in diagnosis. One study identified 9.340 circular RNAs in lung adenocarcinoma [167], but these circular RNAs were generally very poorly expressed in cancerous tissues, with most having fewer than 10 sequenced strands.

Nevertheless, numerous other examples of circular RNAs identified as diagnostic markers have been published. To assess their potential as biomarkers, ROC (receiver operating characteristic) curve analyses have been performed. For example, Wang et al. described a circular RNA, circ-ANXA7 [144], overexpressed in lung adenocarcinomas (*p* < 0.01). Furthermore, a study identified circular RNAs differentially expressed in lung cancer compared to healthy tissues, namely hsa_circ_0077837 and hsa_circ_0001821. This study showed an area under the curve of 0.921 for hsa_circ_0077837 and of 0.863 for hsa_circ_0001821. In addition, circular RNAs whose regulation is specific to a particular subtype of NSCLC have been identified. Hsa_circ_0001073 is specific to adenocarcinomas, with an area under the curve of 0.919, while hsa_circ_0001495 is specific to squamous cell carcinomas, with an area under the curve of 0.965 [145].

A meta-analysis revealed 39 differentially regulated circular RNAs in lung cancer: 28 were overexpressed compared to healthy tissue, while 11 were underexpressed [168]. This study also showed that circular RNA dysregulation is associated with patient prognosis. The overexpression of circular RNAs in tumor cells was associated with a poor prognosis and low overall survival (*p* < 0.00001). Conversely, inhibited expression of circular RNAs was associated with a better prognosis and a better overall survival (*p* = 0.01) [168]. The overexpression of circRNA_102231 and hsa_circ_0013958 in adenocarcinomas was associated with TNM staging (*p* < 0.05 for circRNA_102231, *p* = 0.009 for hsa_circ_0013958) and lymph node metastases (*p* < 0.05 and *p* = 0.006), as well as a poor prognosis [55,146]. Conversely, hsa_circ_100395 was underexpressed in adenocarcinomas and inversely correlated with poor patient prognosis [147].

Recently, a multiparametric analysis using the Nanostring NCounter platform defined a signature of four circRNAs that could be used to diagnose early stages of lung carcinoma with an AUC of 0.981 [154]. However, the use of such a predictive signature still needs to be validated in additional large cohorts prior to clinical use.

In summary, although numerous circRNAs quantified using different techniques have been identified as potential biomarkers in NSCLC tissue biopsies, none of them are currently used in clinics.

### 4.2. Detection of circRNAs in Liquid Biopsies

Tissue biopsies are an essential source of information, but they are more difficult to access. In addition, tumor biopsies are performed at the time of diagnosis and thereby do not provide information on the cancer’s progression during treatment. The patient’s overall health does not always allow for repeated biopsy at the end of treatment or in the event of a relapse. Finally, tumor biopsies do not account for the tumor’s heterogeneity, as they are representative of a localized sample. This is why there is a growing interest in quantifying potential biomarkers in liquid biopsies. Liquid biopsies are samples taken from various body fluids, such as blood, puncture fluids, urine, and saliva. These biopsies are quick and non-invasive, allowing for analysis at different stages of treatment [169].

In blood, numerous biosources have emerged as promising cancer biomarkers, such as circulating tumor cells (CTCs); cell-free tumor DNA (ctDNA), allowing the analysis of mutations or methylation defects; extracellular vesicles (EVs) such as exosomes; and platelets that reflect the transcriptome of tumor cells [170].

Interestingly, more than 1.000 circular RNAs were found in abundance in purified exosomes from human serum in a study conducted by Li et al. in 2015. They also showed the enrichment of these circular RNAs in exosomes compared to the producing cell [171]. The exosome circular RNAs circCCDC134, circFARSA, circ_0047921, circ_0056285, and circ_0007761 are more abundant in the plasma of lung cancer patients compared to the control group (*p* < 0.05 for circCCDC134, *p* < 0.001 for the others). They are therefore potential candidates as diagnostic biomarkers [148,149,151]. Furthermore, the overexpression of circ_0056285 is correlated with an advanced clinical stage (*p* < 0.05) and with the presence of lymph node metastases (*p* = 0.01). Hsa_circ_0069313 is a biomarker overexpressed in the serum of lung cancer patients compared to control groups. Furthermore, it is more discriminating than serum tumor markers (ACE, CA-125, NSE, or CYFRA21-1) in distinguishing benign tumors from NSCLC (AUC = 0.749) [152]. Exosome circular RNAs also provide information on disease prognosis. Hsa_circ_0069313 in the serum is correlated with advanced cancer stage (*p* < 0.001) and lymph node (*p* = 0.001) and distant (*p* < 0.001) metastases [152]. Circ_0008103 and circ_0061407 are expressed at low levels in the serum exosomes of NSCLC patients and associated with diagnostic, advanced pathological staging and distant metastasis [153]. These circRNAs inhibit the proliferation, migration/invasion, and cloning formation abilities of recipient cells to which they are transported. Exosome circular RNAs may also be associated with the response to therapies, such as circVMP1, which is overexpressed in the serum of chemotherapy-resistant patients compared to sensitive patients (*p* < 0.001) [131]. CircVMP1 inhibits miR-524-5p, leading to the overexpression of METTL3 methyltransferase and the cancer stem cell-associated transcription factor SOX2. The exosome transfer of circVMP1 enables the spread of chemoresistance [131].

Multiparametric studies that allow us to define signatures of dysregulated circRNAs rather than monocentric studies based on the detection of a unique circRNA could improve diagnostic potential by increasing accuracy. This has been shown in a study using plasma samples of NSCLC patients analyzed by sequencing. This study revealed the potential of circALG8 and circCAMTA1 as independent biomarkers, with an AUC of 0.7840 and 0.8413, for the diagnosis of early-stage cancer. When the two circRNAs were used combined in a panel, the AUC reached 0.8987 [155], showing the usefulness of a multiparametric panel. Furthermore, the combination of this panel with traditional biomarkers increased the diagnostic accuracy to AUC 0.9236, showing the benefit of adding circRNA quantification to diagnostic procedures. Another study using the nCounter platform analyzed circRNAs extracted from platelets. A signature of five features was found, including two circRNAs and three mRNAs with an AUC of 0.96, to classify early-stage lung cancer [156]. The use of platelets as carriers of biomarkers in cancer has recently emerged and is particularly interesting in the study of circRNAs due to their enrichment in human platelets [156]. The multi-analytical approach combining circRNAs and mRNA improved the AUC, accuracy, and specificity of the diagnostic compared to a monoparametric approach. However, as in solid biopsies, additional clinical validations in larger cohorts and independent validation groups are needed before including circRNA-based biomarker panels in clinical routine.

Thus, although the number of studies highlighting the potential of circRNAs as cancer biomarkers is growing rapidly, the transfer of circRNAs to the clinic requires overcoming certain limitations, such as the cost of the techniques used to quantify them, and the lack of standardization [172].

## 5. Discussion

Although the interest in circular RNAs is extensively growing, notably in the cancer field, there are still relatively few studies investigating circRNAs compared to other components of the cellular transcriptome, such as miRNAs or lncRNAs [26]. Until now, most of the functions of circRNAs have been related to their ability to sponge miRNAs. In addition, circRNAs are still mainly considered non-coding RNAs. We must undoubtedly reconsider these claims in the future. The relevance of the miRNA sponge model of circRNAs is now being questioned [37]. In addition, an increasing number of studies have reported coding functions for circRNAs, whose products could play important roles yet to be explored [49]. In particular, the ability of circRNAs to generate neo-antigens in cancer through their translation opens new avenues for therapeutic purposes [46,173]. Nevertheless, although circRNA translation amplifies the number of neo-epitopes, their reliability depends on the amount of circRNA translation, the immunogenicity of the neo-antigens, and their ability to bind to HLA [47]. Thus, the challenge before using these neo-antigens in immunotherapy is to identify circRNA-encoded peptides that can induce sufficient immune responses. The stability and half-lives of the proteins translated from circRNAs must also be taken into account for the development of these new therapeutic strategies [174].

In NSCLC, circRNAs affect the behavior of cancer cells by deregulating their apoptosis, their migration and invasion capacities, their proliferation, their plasticity, and the expression of certain oncogenes or tumor suppressor genes [175]. In addition, circRNAs impact the crosstalk between tumor cells and cells from the tumor microenvironment, which involves the regulation of the secretome of cancer cells, the modulation of the expression of surface markers, and the secretion of circRNAs inside extracellular vesicles, allowing the transfer of these circRNAs between cells [176,177,178]. In particular, circRNAs can reshape the tumor immune microenvironment, a reshaping that contributes to the either pro- or anti-tumoral functions of these circRNAs, and can regulate the response to immunotherapies. As a whole, these data support the need to deepen our knowledge regarding the role of circRNAs in inter-cellular communication [94,177,179,180].

In this review, we do not discuss the therapeutic strategies developed based on circRNA targeting. Indeed, taking into account their roles as miRNAs or RBP sponges, circRNAs have emerged as promising therapeutic targets. These therapeutic strategies are based on inhibiting circRNA expression by siRNA transfection, using antisense oligonucleotides, or by gene editing [13]. Engineered circRNAs can also be used in clinical applications as carriers of peptides for drug release or vaccine delivery. CircRNA-loaded DCs are also under investigation and show promising results in pancreatic cancer [181]. However, the use of circRNAs as therapeutic strategies is still in its infancy and faces many challenges, such as overcoming off-target effects.

CircRNAs’ stability and resistance to RNase degradation make them promising biomarkers for cancer diagnosis and prognosis, including in lung cancer, and consistently, numerous studies are now assessing their potential in solid or liquid biopsies [182]. However, despite some circRNAs having demonstrated significant relevance in the diagnosis of lung cancer, none are currently used in clinical practice. Again, before using circRNA detection/quantification for therapeutic purposes, we clearly need to overcome challenges such as the lack of standardized methodology to quantify circRNAs with sufficient sensitivity and precision. In addition, future studies need to test circRNAs’ value as biomarkers in multicentric clinical trials with accurate and precise quantification [183]. In this setting, the development of new approaches like single-cell RNA sequencing (scRNA-seq), spatial transcriptomics, and multi-omics could help fill the gap in our understanding of circRNAs’ biology and functions in lung cancer [184].

## 6. Conclusions

There is growing interest in circular RNAs in oncology, particularly in NSCLC. They are involved in tumorigenesis; the development, migration, and invasion capacity of cancer cells; and their resistance to therapies. They also play a role in regulating the tumor microenvironment. Their circular structure makes them more resistant to degradation, and their specific back-junction sequence makes it possible to develop molecular techniques for detecting and quantifying circular RNAs, not only in tumor tissues but also in liquid biopsies. Their use as markers for diagnosis, prognosis, or resistance to therapy requires the standardization of quantitative techniques and validation in multicenter studies.

## Figures and Tables

**Table 1:** Examples of circular RNAs serving as potential biomarkers (diagnosis, prognosis) in lung cancer. The 23 circular RNAs implicated in resistance to therapies are shown in bold.

CircRNA	Samples	Biomarkers Type	Correlation with Clinico-Pathological Parameters	Reference
CiRS-7	Tissues	Diagnostic Prognostic	– TNM Stage – Lymph node metastasis	[115]
CircHIPK3	Tissues	Diagnostic		[116]
**CircUSP7**	**Tissues** **Plasma**	**Diagnostic** **Pronostic** **Resistance to therapies**	– **Tumor size** – **Lymph node metastasis** – **Overall survival**	[52]
Hsa_circ_0013958	Tissues Plasma	Diagnostic	– TNM Stage – Lymph node metastasis	[55]
CircRACGAP1	Tissues	Diagnostic		[56]
CircITCH Under expression	Tissues	Diagnostic		[57]
CircERBB2IP	Serum	Diagnostic	– TNM Stage – Lymph Node Metastasis – Tumor Size	[59]
CircLIMK1-005	Tissues	Diagnostic Pronostic		[60]
CircPVT1	Tissues	Diagnostic Prognostic	– Tumor size – TNM stage – Overall survival	[61]
CircPUM1	Tissues	Diagnostic	– TNM stage	[62]
Hsa_circ_0020123	Tissues	Diagnostic Prognostic	– Differentiation – TNM Stage – Lymph node metastasis	[63]
CircPTK2 Under expression	Tissues	Diagnostic	– Metastasis	[67]
CircDLG1	Tissues	Diagnostic	– Overall survival	[69]
CircVAPA	Tissues	Diagnostic	– Overall survival	[70]
C190	Tissues	Diagnostic Prognostic	– Advanced stage	[71]
Circ-ENO1	Tissues	Diagnostic		[73]
CircZNF707	Tissues	Diagnostic		[74]
CircDCUN1D4 Under expression	Tissues	Diagnostic	– Advanced stage – Lymph node metastasis – Overall survival	[75]
Hsa_circ_0003222	Tissues	Diagnostic Prognostic	– TNM Stage – Metastasis – Survival	[77]
hsa_circ_0077837	Tissues	Diagnostic	– Tumor size – TNM stage – Lymphatic metastases – Overall survival	[82]
Circ_0000064	Tissues	Diagnostic	– Tumor size – TNM stage – Lymph node metastasis	[83]
circCENPF	Tissues	Prognosis	– Immune microenvironment infiltration – Pathological stage	[89]
Circ_BBS9 Under expression	Tissues	Early diagnostic Prognosis	– Poor prognosis	[90]
CircFNDC3B	Tissues	Diagnostic		[92]
**cEMSY**	**Tissues**	**Response to therapy**	– **Response to ICIs**	[93]
CircCHST15	Tissues	Diagnostic		[95]
CircRNA-002178	Tissues Serum	Diagnostic	– Early stage	[96]
Circ_001678	Tissues			[97]
CircIGF2BP3	Tissues		– Immune microenvironment infiltration	[100]
circ_0003528	Tissues	Diagnostic Prognosis	– Worse prognosis	[101]
Circ_0092012	Tissues	Diagnostic	– Tumor size – Lymph node metastasis – TNM grade	[102]
CircSHKBP1	Tissues Serum	Diagnostic	– TNM stage – Lymphatic metastasis – Overall survival	[105]
**CircZNF451**	**Tissues** **Serum**	**Prognosis** **Response to therapy**	– **Progressive disease** – **Poor prognosis** – **Sensitivity to PD1 blockage**	[106]
CircPLEKHM1	Tissues	Prognosis	– Shorter Overall survival – Shorter Metastasis-free survival	[107]
CircATP9A	Tissues	Diagnostic	– Elevated stage	[109]
Circ_0072083	Tissues	Diagnostic		[117]
Circ_0007385	Tissues	Diagnostic	– Advanced stage – Overall survival	[118]
**Circ_PIP5K1A**	**Tissues**	**Resistance to therapies**	– **Cisplatin resistance**	[119]
**Circ_0010235**	**Tissues**	**Resistance to therapies**	– **Cisplatin resistance**	[120]
Hsa_circ_0085131	Tissues	Diagnostic Prognostic	– Overall survival – Recurrence	[121]
**CircRNA_100565**	**Tissues**	**Diagnostic** **Pronostic** **Resistance to therapies**	– **TNM Stage** – **Lymph Node Metastasis** – **Chemoresistance** – **Overall Survival**	[122]
CircLDB2	Tissues	Diagnostic	– TNM Stage – Lymph node metastasis	[123]
**Circ_0000079** **Under expression**	**Tissues**	**Diagnostic** **Resistance to therapies**	– **Overall survival** – **Chemoresistance**	[124]
**CircHUWE1**	**Tissues**	**Diagnostic** **Resistance to therapies**		[125]
**Hsa_circ_0004015**	**Tissues**	**Diagnostic** **Resistance to therapies**	– **TNM Stage** – **Differentiation** – **Advanced Stage** – **Low Survival Rate** – **Cisplatin resistance**	[126,127]
**Circ_0110498**	**Tissues**	**Diagnostic** **Resistance to therapies**	– **Chemoresistance**	[128]
**Circ_0076305**	**Tissues** **Serum**	**Diagnostic** **Resistance to therapies**	– **Chemoresistance**	[129]
Circ_103615	Tissues	Diagnostic	– Tumor size – TNM stage – Metastasis – Overall survival	[130]
**CircVMP1**	**Serum**	**Resistance to therapies**	– **Chemoresistance**	[131]
Hsa_circ_0001946	Tissues	Diagnostic		[132]
**CircRNA_102481**	**Serum**	**Resistance to therapies**	– **TNM stage** – **Tumor differentiation status** – **Brain metastasis** – **Shorter overall survival** – **Shorter Progression-free survival** – **EGFR-TKI resistance**	[133]
**CircSETD3**	**Tissues**	**Resistance to therapies**	– **Osimertinib resistance**	[134]
**Circ_0002130**	**Tissues** **Serum**	**Diagnostic** **Resistance to therapies**	– **Osimertinib resistance**	[135]
**Has_circ_0078465**		**Resistance to therapies**	– **Osimertinib resistance**	[136]
**CircSPINT2**		**Resistance to therapies**	– **Osimertinib resistance**	[137]
F-circEA1		Diagnostic	– EML4-ALK1 positive	[138,139]
**CircFGFR1**	**Tissues**	**Diagnostic** **Pronostic** **Resistance to therapies**	– **Tumor size** – **Differentiation** – **Lymph node metastasis** – **Overall survival** – **Relapse-free survival** – **Resistance to anti-PD-1**	[140]
**Circ_CELF1**	**Tissues**	**Resistance to therapies**	– **Malignant characteristics** – **Poor outcome** – **Anti-PD-1 resistance**	[141]
**CircHMGB2**	**Tissues**	**Pronostic** **Resistance to therapies**	– **Tumor size** – **Clinical stage** – **Lymph node metastasis** – **Poor overall survival** – **Postoperative recurrences rates** – **Anti-PD-1 resistance**	[142]
**Circ_PPAPDC1A**	**Tissues**	**Diagnostic** **Resistance to therapies**	– **Shorter PFS** – **ICI resistance**	[143]
Circ-ANXA7	Tissues	Diagnostic Prognostic	– TNM stage – Lymph node metastasis – Poor prognosis	[144]
Hsa_circ_0001821	Tissues	Diagnostic		[145]
Hsa_circ_0001073	Tissues	Diagnostic	– Adenocarcinoma	[145]
Hsa_circ_0001495	Tissues	Diagnostic	– Squamous cell carcinoma	[145]
CircRNA_102231	Tissues	Diagnostic	– TNM Stage – Lymph Node Metastasis – Survival	[146]
Hsa_circ_100395 Under expression	Tissues	Prognostic	– TNM Stage – Lymph node metastasis – Metastasis – Poor prognosis	[147]
CircCCDC134	Tissues Serum	Diagnostic		[148]
CircFARSA	Tissues Plasma	Diagnostic		[149,150]
Circ_0047921 Under expression	Serum	Diagnostic		[151]
Circ_0056285	Serum	Diagnostic	– TNM Stage – Lymph node metastasis	[151]
Circ_0007761	Serum	Diagnostic		[151]
Hsa_circ_0069313	Serum	Diagnostic	– Advanced stage – Lymph node metastasis – Metastasis	[152]
Circ_0008103 Under expression	Serum	Diagnostic	– Advanced pathological staging – Distant metastasis	[153]
Circ_0061407 Under expression	Serum	Diagnostic	– Advanced pathological staging – Distant metastasis	[153]
4 circRNA signature: CircEPB41L2 CircSOX13 CircBNC2 CircCORO1C	Tissue	Diagnostic		[154]
circALG8 + circCAMTA1	Tissue Plasma	Diagnostic	– TNM Stage	[155]
2 circRNAs CircSLC8A1 CircCHD9 + 3 mRNAs PSMB9 RUNX1 LILRB1	Blood Platelets	Diagnostic		[156]

## Data Availability

No new data were created or analyzed in this study. Data sharing is not applicable to this article.

## Abbreviations

The following abbreviations are used in this manuscript:

circRNA	Circular RNA
RNA	Ribonucleic Acid
BSJ	Back Splicing Junction
RBP	RNA-binding protein
snRNA	small nuclear RNA
dsRBD	double-stranded RNA binding domain
NF	Nuclear Factor
QKI	Quaking
ecircRNA	Exonic circular RNA
ciRNA	Circular intronic RNA
elciRNA	exon-intron circular RNA
f-circRNA	Fusion circular RNA
mRNA	Messenger RNA
rt-circRNA	Read-through circRNA
snRNP	small nuclear ribonucleoprotein
DNA	Deoxyribonucleic Acid
circZNF609	Circular Zinc finger protein 451
circEIF3J	Circular Eukaryotic translation initiation factor 3 subunit J
YBX1	Y box binding protein 1
*FZD7*	Frizzled-7
circZN	Circular Zinc finger protein
CKAP5	Cytoskeleton Associated Protein 5
*DNMT*	DNA methyltransferase
miRNA	Micro RNA
*CDR1*	Cerebellar degeneration-related protein 1
AGO2	Argonaute
HIPK3	homeodomain interacting protein kinase 3
CDK	cyclin-dependent kinases
MDM2	Mouse double minute 2 homolog
circRHOT1	Circular Ras Homolog Family Member T1
*NRF2*	Nuclear factor erythroid 2-related factor 2
TIP60	Tat-interactive protein 60
NSUN2	NOP2/Sun domain family, member 2
IGF2BP2	Insulin Like Growth Factor 2 MRNA Binding Protein 2
*HMGA2*	HMGA2 high mobility group AT-hook 2
circYAP	Circular Yes-associated protein
eIF4G	Eukaryotic Translation Initiation Factor
PABP	Poly(A)-binding protein
eIF	eukaryotic Initiation Factors
IRES	Internal Ribosome Entry Site
MAPK	Mitogen-Activated Protein Kinase
m6A	Methylation at the sixth nitrogen atom of adenosine
YTHDF3	YTH N6-Methyladenosine RNA Binding Protein F3
EGFR	Epidermal Growth Factor Receptor
circRASSF2	Circular Ras Association Domain Family Member 2
MMP1	Matrix metalloproteinase 1
USP7	Ubiquitin Specific Peptidase 7
SHP2	SRC region homologue 2
PD-L1	Programmed cell Death Ligand 1
PD-1	Programmed cell death protein 1
SCLC	Small Cell Lung Cancer
NSCLC	Non-Small Cell Lung Cancer
ALK	Anaplastic Lymphoma Kinase
circRACGAP1	Circular Rac GTPase-activating protein 1
ITCH	Itchy E3 Ubiquitin Protein Ligase
DVL2	Dishevelled Segment Polarity Protein 2
CircERBB2IP	Circular Erbb2 Interacting Protein
*PSAT1*	Phosphoserine aminotransferase 1
LUAD	Lung adenocarcinoma
RPA1	Replication Protein A1
LIMK1	LIM domain kinase 1
Bcl-2	B-cell lymphoma 2
PUM1	P53 Upregulated Modulator of Apoptosis
ZEB1	Zinc finger E-box-binding homeobox 1
EZH2	Enhancer of zeste homolog 2
CORO1C	Coronin-1C
EMT	Epithelial to Mesenchymal Transition
TGF-β	Transforming Growth Factor-β
PTK2	Protein Tyrosine Kinase 2
CircDLG1	Circular Discs large homolog 1
circVAPA	Circular VAMP-Associated Protein A
ENO1	Enolase 1
PFKM	Phosphofructokinase Muscle
CircDCUN1D4	Circular Defective In Cullin Neddylation 1 Domain Containing 4
*TXNIP*	Thioredoxin Interacting Protein
CSC	Cancer stem cell
SOX2	SRY-Box Transcription Factor 2
OCT4	Octamer-binding transcription factor 4
ALDH1A1	Aldehyde Deshydrogenase 1 type H1
PHF21B	PHD Finger Protein 21B
TKI	Tyrosine kinase inhibitor
*BCL6B*	B-cell CLL/lymphoma 6 member B transcription repressor
METTL14	Methyltransferase
PTEN	Phosphatase and TENsin homolog
TME	tumor microenvironment
ECM	extracellular matrix
MMP	Matrix metalloproteinase
VEGF	Vascular Endothelial Growth Factor
SRSF1	Serine/arginine-rich splicing factor1
HIF-1	Hypoxia-inducible factor 1
TIME	Tumor Immune MicroEnvironment
NK	Natural Killer
IFIT3	Interferon Induced Protein with Tetratricopeptide Repeats 3
CXCL	chemokine (C-X-C motif) ligand
CD	Cluster differenciation
CCL5	Chemokine ligand 5
IFN-β	Interferon- β
circNDUFB2	Circular NADH:ubiquinone oxidoreductase subunit B2
STAT1	signal transducer and activator transcription 3
IS	Immune System
CircCHST15	Circular Carbohydrate Sulfotransferase 15
IL	Interleukine
HSP90A	Heat Shock Protein 90A
PKP3	Plakophilin 3
FXR1	Fragile X Mental Retardation, Autosomal Homolog 1
OTUB1	OTU Deubiquitinase, Ubiquitin Aldehyde Binding 1
TAM	Tumor-Associated Macrophages
SHKBP1	SH3KBP1 Binding Protein 1
PKM2	Pyruvate Kinase M2
HK2	Hexokinase 2
TRIM56	Tripartite Motif Containing 56
ELF4-IRF4	E74-like factor 4 -Interferon regulatory factor 4
OSMR	Oncostatin M Receptor
JAK	The Janus kinase
TREM2	Triggering Receptor Expressed On Myeloid Cells 2
hnRNP	Heterogeneous nuclear Ribonucleoproteins
DC	Dendritic cell
GDF15	Growth/Differentiation Factor 15
MALAT-1	Metastasis Associated Lung Adenocarcinoma Transcript 1
NF-κB	Nuclear Factor kappa B
Snx5	Sorting Nexin 5
CircAEBP2	Circulaire AE Binding Protein 2
circFSCN1	Circulaire Fascin Actin-Bundling Protein 1
MHC	Major histocompatibility complex
CBLL1	Cbl proto-oncogene like 1
HMGB1	High Mobility Group Box 1
ROCK1	Rho Associated Coiled-Coil Containing Protein Kinase 1
ATG7	Autophagy Related 7
LDB2	(LIM Domain Binding 2
LIMCH1	LIM And Calponin Homology Domains 1
PRCKI	Protein-kinase C
TNFAIP8	TNF Alpha Induced Protein 8
RBBP4	RB binding protein 4
GOT1	Glutamic-Oxaloacetic Transaminase 1
NER	nucleotide excision repair
PDPK1	3-Phosphoinositide Dependent Protein Kinase 1
IGF1R	Insulin-like Growth Factor 1 Receptor
EML4-ALK	Echinoderm Microtubule-associated protein-Like 4-Anaplastic Lymphoma Kinase
SHP2	SRC homologous region 2
TCR	T cell receptor
ICI	immune checkpoint inhibitor
ROC	Receiver Operating Characteristic
CTC	circulating tumor cell
ctDNA	circulating tumor DNA
EV	Extracellular vesicles
RT-qPCR	Reverse Transcription quantitative Polymerase Chain Reaction
ddPCR	Digital droplet PCR
siRNA	Small interfering RNA
scRNA-seq	single cell RNA-sequencing
